# Actions on childhood voice in public schools: a scoping review

**DOI:** 10.1590/2317-1782/e20240367en

**Published:** 2026-01-26

**Authors:** Luciana Tavares Sebastião, Nelma Ellen Zamberlan-Amorim, Fabiana Zambon, Vanessa Veis Ribeiro, Mara Behlau

**Affiliations:** 1 Centro de Estudos da Voz – CEV - São Paulo (SP), Brasil.; 2 Hospital das Clínicas, Faculdade de Medicina de Ribeirão Preto - Ribeirão Preto (SP), Brasil.; 3 Sindicato dos Professores de São Paulo – SINPRO-SP - São Paulo (SP), Brasil.; 4 Faculdade de Ciências e Tecnologias em Saúde, Universidade de Brasília – UnB - Brasília (DF), Brasil.

**Keywords:** Voice, Dysphonia, Preschool Children, Education Primary and Secondary, Policy Public, Speech, Language and Hearing Sciences

## Abstract

**Purpose:**

To map childhood voice interventions developed in Brazilian public schools and analyze them according to components of the School Health Program (SHP).

**Research strategies:**

This was a scoping review, structured according to the methodological guidelines of the Joanna Briggs Institute (JBI). The research question was, “What childhood voice interventions are developed in Brazilian public schools, and which SHP components are included in these interventions?”. An electronic search was conducted in the MEDLINE, LILACS, Scopus, Web of Science, Embase, and Cochrane databases, along with a manual search of gray literature, citation lists, and expert consultations.

The search strategies consisted of free and indexed terms related to the topic and adapted for each source of evidence.

**Selection criteria:**

The review included studies involving schoolchildren up to 12 years old with voice interventions developed by speech-language-hearing pathologists in public schools, published after 2007. It extracted data regarding the publication, the sample, and childhood voice interventions according to the SHP components.

**Data analysis:**

Data analysis was descriptive.

**Results:**

4,087 studies were found, and 27 were selected. The most common interventions involved students, followed by parents and teachers. Twenty-six studies included assessments of students' health conditions, and one included health promotion and disease prevention actions in an online course for parents and students. The most common assessment procedure was auditory-perceptual evaluation.

**Conclusion:**

Studies on childhood voice in schools are scarce and prioritize assessment of vocal health conditions.

## INTRODUCTION

Public policy is a set of government actions with specific objectives, whose activities influence the lives of citizens^(1)^. The right to health reaches citizens through public policies that aim to provide comprehensive care, with an emphasis on preventive activities, social control, and better health indicators^(2-4)^. There is a global incentive for greater investment in promoting health and improving the population's quality of life^(5)^.

Speech-language-hearing (SLH) pathology, as a health profession, supports this perspective through the recent planning of the American SLH Association (ASHA) It combines efforts to ensure that, by 2030, the profession will expand its emphasis on actions aimed at improving the quality of life in healthy populations, addressing the determinants of communication health and healthy lifestyles related to human communication^(6)^.

The School Health Program (SHP) is a Brazilian interministerial policy, established by Presidential Decree No. 6,286, of December 5, 2007^(7)^. It is currently structured into three components: Assessment of schoolchildren's health conditions (Component I); Health promotion and disease prevention (Component II); and Training (Component III). The latter involves training program managers, education and health teams, and young protagonists for the SHP^(8,9)^.

It is estimated that around 12.8% of children enrolled in Brazilian public elementary schools have voice disorders^(10)^. Vocal disorders can have a negative impact on the quality of life of students by hindering efficient communication and participation in social and educational activities^(11,12)^.

Children's voices have peculiarities resulting from the neuromuscular immaturity of the vocal tract and the shape of the laryngeal cartilages. Vocal characteristics such as hoarseness, breathiness, and mild instability are expected in children due to the immature vocal ligament, the lack of differentiation of the vocal fold layers, the high vascularization of the vocal folds with a tendency to edema, the size and position of the vocal folds resulting from the shape of the laryngeal cartilages in childhood, and neuromuscular immaturity. Children’s voices do not differ according to sex, and there may be slight variations due to resonance in the vocal tract^(13)^.

Schoolchildren with voice disorders may present with complaints such as hoarseness, pain, throat clearing, and coughing when speaking and singing, difficulty reading aloud, or singing during games and plays. They may also experience frustration, anger, embarrassment, and dissatisfaction with their voice^(14)^. Nevertheless, only about 23% of children with voice disorders seek SLH therapy^(15)^.

These data confirm the need for SHP actions to promote vocal health and prevent, identify, treat, and monitor voice disorders in schools.

Therefore, it is important to map child voice actions being developed in Brazilian public schools and classify them based on SHP components. It is believed that these data will provide a greater understanding of the state of the art in SHP implementation among Brazilian schoolchildren, as well as evidence that can inform discussions about the limits and needs for investment in this area, aiming to reduce these students’ developmental risks and improve their quality of life.

Review studies on voice actions developed with public school students are quite scarce and, in general, seek to analyze vocal and behavioral aspects, not aiming, as in this article, to characterize the voice actions developed in schools considering public health policies^(16,17)^.

This scoping review aimed to map actions on children’s voices developed in Brazilian public schools and classify the aspects addressed according to SHP components.

## METHODS

This scoping review followed the Joanna Briggs Institute methodology^(18)^ and was written according to the PRISMA-ScR recommendations. The protocol for this scoping review was registered with the Open Science Framework (doi: 10.17605/OSF.IO/UE94W).

The research question was guided by the acronym P - Participant; C - Concept; and C - Context (PCC), namely: P - children; C - SHP components present in voice-related actions; and C - public schools in Brazil. The research questions that guided the review were, “What childhood voice-related actions are developed in public schools in Brazil? Which SHP components are included in these actions?”.

The search was conducted electronically and manually. The electronic search was conducted in the LILACS (VHL), MEDLINE (PubMed), EMBASE, Web of Science, SCOPUS, and Cochrane Library databases. The manual search was conducted through citation mapping, gray literature (Google Scholar, medRxiv, and ProQuest), and expert consultation (mapping publications by corresponding authors with more than three articles selected in the electronic or manual search).

The search strategy was developed based on indexed keywords (Medical Subject Headings – MeSH and Emtree terms) and free terms related to the PCC. A search strategy was initially developed for MEDLINE, later adapted for other sources of evidence (Appendix A).

Studies involving schoolchildren up to 12 years old with voice interventions developed by SLH pathologists in Brazilian public schools, published after 2007, were selected.

Inclusion and exclusion criteria were established for study selection. It included articles involving schoolchildren aged 0 to 12 years and interventions on children's voices conducted by SLH pathologists in Brazilian public schools. Literature reviews, conference proceedings, and undergraduate final papers; articles on interventions involving the voices of adolescents, adults, or older adults (older than 12 years); studies with samples composed solely of schoolchildren from private schools; studies conducted in countries other than Brazil; duplicate studies; studies with duplicate samples; and studies accepted for publication before 2007 were excluded. The publication year filter was used because the SHP was established in December 2007. No language filter was used. The age cutoff of 12 years was based on Law No. 8,069, of July 13, 1990, which establishes the Statute of Children and Adolescents, considering as a child anyone up to 12 years of age^(19)^.

Study selection followed three steps: removing duplicates; reading the title and abstract, applying the inclusion criteria; and reading the full text, applying the exclusion criteria. The electronic search was performed on the Rayyan website. This process will be described in the results, using the PRISMA-ScR selection flowchart. Two independent authors performed the study selection, and disagreements were resolved by consensus.

The reviewers were preliminarily calibrated before the selection began, using 25 articles. To pass the calibration, the reviewers needed to obtain at least 0.7 Cohen's kappa coefficient of agreement. Following calibration, the coefficient of agreement was 1.0. Cohen's kappa values ​​between 0.81 and 1.0 indicate near-perfect agreement^(20)^. The search was conducted in June 2024, and the selection was conducted between June and July 2024.

The authors developed a specific instrument for this study’s data extraction, encompassing publication (authors, year of publication, state where the study was conducted, journal); sample characteristics (sample size, level and type of education, participants’ age and sex); and characteristics of the actions developed in schools, according to SHP components (assessment of health status; health promotion and disease prevention; and training). The authors of this scoping review classified the actions in the selected studies according to the description of the three current SHP components^(8,9)^.

Two independent reviewers extracted data, and disagreements were resolved by consensus. If additional data or confirmation of information was necessary, up to three attempts to contact the corresponding author via email were scheduled.

Data were analyzed descriptively, using simple and relative frequencies. The results are presented in the form of charts, graphs, tables, and figures.

## RESULTS

**Figure 1:**
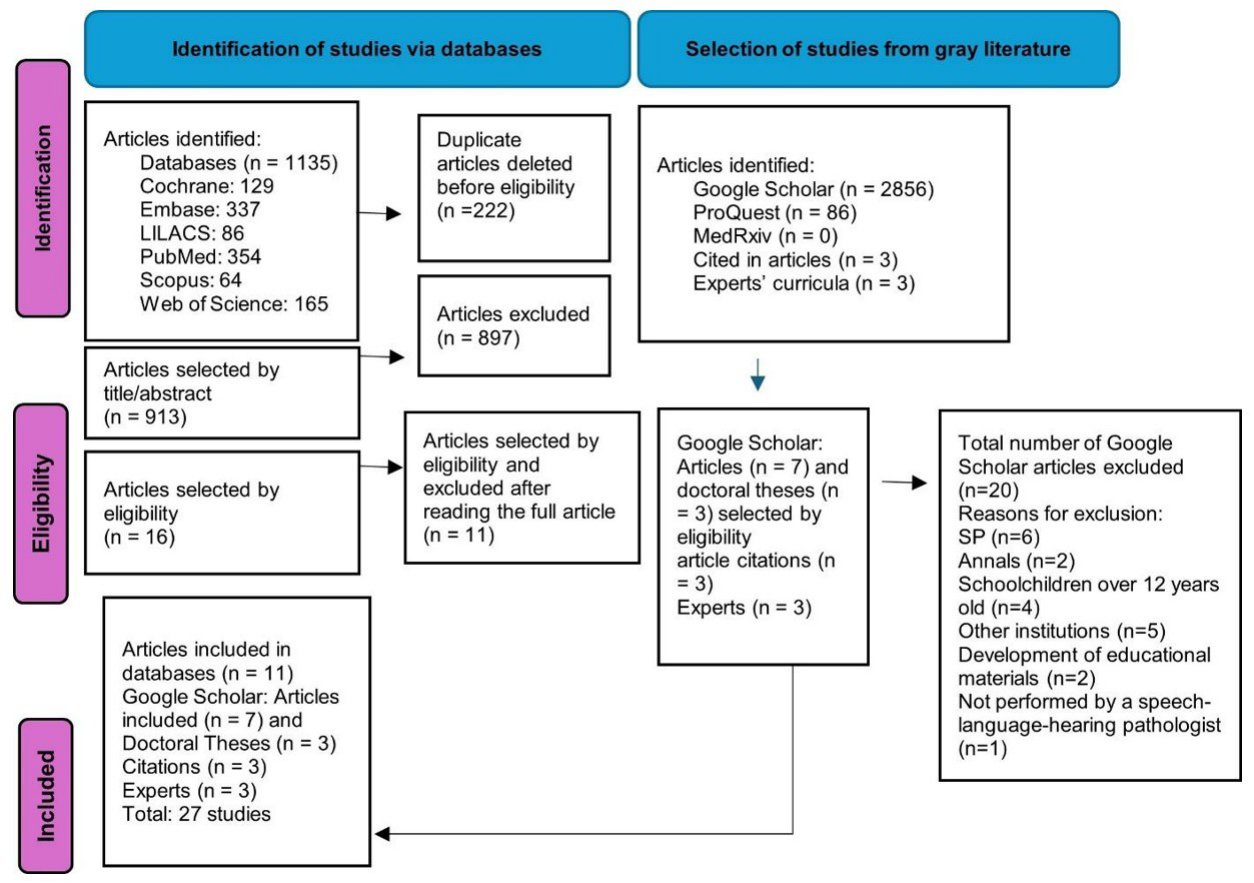
Study selection flowchart

Source: Page MJ, et al.^(21)^

Figure 1 presents the studies identified in the electronic and manual searches. The electronic search initially identified 1,135 studies. Of these, 222 studies were removed due to duplication, and 897 did not meet the inclusion criteria. The first selection phase included 16 articles based on title and abstract reading. The second phase excluded five articles – three because the procedures were not conducted in public schools, one for presenting only a situational assessment of the school, and one for involving a database of teachers' voices. Thus, 11 studies were selected from the electronic search.

**Caption:** n = absolute frequency; SP = senior project

The manual search identified 2,856 studies in Google Scholar and 86 in ProQuest. Of these, 30 were included based on title and abstract reading. The full review excluded 20 studies – six for being final year papers; four for including students over the age of 12; five for not having been conducted with public school students; two because they were published in conference proceedings; two because they only presented the development of educational materials; and one because it was not conducted by an SLH pathologist. Thus, 10 studies were selected at this stage.

During the manual search, three studies were included through citation mapping, and three through expert consultation.

Thus, 27 studies were selected for this scoping review, including 24 articles and three doctoral theses.

Regarding publication characteristics (Table 1), 14.8% (n = 4) of the studies were published in the last 5 years (2020 to 2024). The years with the highest publication frequency were 2012 and 2016 (n = 3; 11.1% each). The studies originated from research groups in four regions and eight Brazilian states, primarily from Southern Brazil (n = 12; 44.4%) and the state of Rio Grande do Sul (n = 9; 33.3%). Among the 24 articles, 75% (n = 18) were published in national journals, most frequently in the Revista CEFAC (n = 7; 38.9%).

**Table 1:** Publication characteristics

Authors	State	year	Journal
Cielo and Cappellari^(22)^	RS	2008	Revista Brasileira de Otorrinolaringologia
Cappellari and Cielo^(23)^	RS	2008	Revista Brasileira de Otorrinolaringologia
Sales et al.^(24)^	SE	2010	Journal of Voice
Silva et al.^(25)^	PR	2012	Jornal da Sociedade Brasileira de Fonoaudiologia
Sales et al.^(26)^	SE	2013	Journal of Voice
Sales et al.^(27)^	SE	2013	Journal of Voice
Pascotini et al.^(28)^	RS	2015	Distúrbios da Comunicação
Pascotini et al.^(29)^	RS	2016	Journal of Voice
Marangon et al.^(10)^	SP	2018	Revista CEFAC
Aires et al.^(30)^	RS	2019	Journal of Voice
Hoffmann and Cielo^(31)^.	RS	2021	Journal of Voice
Schott et al.^(32)^	RJ	2009	Revista CEFAC
Braga et al.^(33)^	MG	2009	Revista CEFAC
Oliveira et al.^(12)^	MG	2011	Jornal da Sociedade Brasileira de Fonoaudiologia
Paixão et al.^(34)^	PR	2012	Revista CEFAC
Souza et al.^(35)^	MG	2017	CoDAS
Silva and Souza^(36)^	RN	2020	Revista Brasileira de Ciências da Saúde
Reis et al.^(37)^	PE	2021	Revista CEFAC
Tavares et al.^(38)^	SP	2011	Brazilian Journal of Otorhinolaryngology
Guerra et al.^(39)^	PE	2014	Distúrbios da Comunicação
Paixão et al.^(40)^	PR	2015	Distúrbios da Comunicação
Pascotini et al.^(41)^	RS	2014	Revista Brasileira de Qualidade de Vida
Pascotini et al.^(42)^	RS	2016	Revista CEFAC
Cielo et al.^(43)^	RS	2016	Revista CEFAC
Maia^(44)^	MG	2012	Doctoral thesis (UFMG)
Nunes^(45)^	MG	2017	Doctoral thesis (UFMG)
Oliveira^(46)^	SP	2023	Doctoral thesis (Unesp Marília)

Regarding the sample characteristics (Chart 1), students were present in 92.6% (n = 25), parents in 33.3% (n = 9), and teachers in 7.4% (n = 2) of the studies. The sample sizes ranged from 15 to 2,000 students and from 15 to 198 parents. One of the studies included one teacher, and the other did not report the number of participating teachers. The students’ age ranged from 4 to 12 years, and 85.2% (n = 23) of the studies included students of both sexes. It is worth noting that 14.8% (n = 4) of the studies did not report the sex of the participants. Studies with elementary school students were more frequent (n = 22; 81.5%). Moreover, studies with students exclusively from public schools predominated (n = 20; 74%), although some studies had samples with students from both public and private schools (n = 7; 26%).

**Chart 1:** Sample characteristics

Authors and year	Participants			Sample Size	Age (years)	Sex	Education Level	School Type
	Children	Parents	Teachers					
Cielo and Cappellari, 2008^(22)^	**º**			23 children	4:0 to 6:8	Both sexes	Preschool	Public Private
Cappellari and Cielo, 2008^(23)^	**º**			23 children	4:0 to 6:8	Both sexes	Preschool	Public Private
Sales et al., 2010^(24)^	**º**			600 children	7 to 10	Both sexes	Elementary school	Public
Silva et al., 2012^(25)^	**º**			38 children	7 to 11	Both sexes	Elementary school	Public
Sales et al., 2013^(26)^	**º**			196 children	7 to 10	Both sexes	Elementary school	Public
Sales et al., 2013^(27)^	**º**			60 children	7 to 10	Both sexes	Elementary school	Public
Pascotini et al., 2015^(28)^		**º**		104 parents	8 to 12	Both sexes	Elementary school	Public
Pascotini et al., 2016^(29)^	**º**			82 children	8 to 10	Both sexes	Elementary school	Public
Marangon et al., 2018^(10)^	**º**			250 children	6 to 9	Both sexes	Elementary school	Public
Aires et al., 2019^(30)^	**º**			154 children	8 to 10	Both sexes	Elementary school	Public Private
Hoffmann and Cielo, 2021^(31)^	**º**			115 children	4:7 to 7:11	Not reported	Elementary school	Public Private
Schott et al., 2009^(32)^	**º**			122 children	6:0 to 8:11	Both sexes	Elementary school	Public
Braga et al., 2009^(33)^	**º**			100 children	6 to 8	Both sexes	Elementary school	Public
Oliveira et al., 2011^(12)^	**º**			70 children	6 to 10	Both sexes	Elementary school	Public
Paixão et al., 2012^(34)^	**º**	**º**		50 children 100 parents	6 to 12	Both sexes	Elementary school	Public
Souza et al., 2017^(35)^	**º**			420 children	6 to 10	Not reported	Elementary school	Public Private
Silva and Souza, 2020^(36)^	**º**			63 children	8 to 12	Both sexes	Elementary school	Public
Reis et al., 2021^(37)^	**º**			31 children	6 to 7	Both sexes	Elementary school	Public
Tavares et al., 2011^(38)^	**º**			2.000 children	4 to 12	Both sexes	Preschool Elementary school	Public Private
Guerra et al., 2014^(39)^		**º**	**º**	20 parents 1 teacher	4 to 6	Both sexes	Preschool	Public
Paixão et al., 2015^(40)^	**º**	**º**		50 children 100 parents	6 to 12	Not reported	Elementary school	Public
Pascotini et al., 2014^(41)^	**º**	**º**		83 children	8 to 12	Both sexes	Elementary school	Public
Pascotini et al., 2016^(42)^	**º**			82 children	8 to 10	Both sexes	Elementary school	Public
Cielo et al., 2016^(43)^	**º**	**º**		102 children	8 to 12	Both sexes	Elementary school	Public
Maia, 2012^(44)^	**º**	**º**	**º**	198 children 198 parents teachers NI	7 to 10	Both sexes	Elementary school	Public
Nunes, 2017^(45)^	**º**	**º**		420 children	6 to 10	Both sexes	Elementary school	Public Private
Oliveira, 2023^(46)^	**º**	**º**		19 children 15 parents	4 to 11	Not reported	Preschool Elementary school	Public

**Figure 2:**
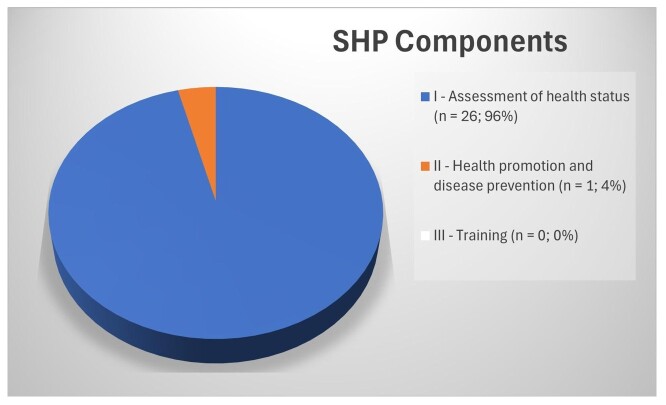
Characterization of the School Health Program components addressed in the studies

Only two of the three SHP components were present in the studies, with emphasis on Component I — assessment of students' health status (Figure 2).

Chart 2 shows that, among the 26 studies that assessed the schoolchildren’s health status, 46.2% (n = 12) involved questionnaires or interviews with parents or guardians of students to collect information on the general health of schoolchildren (n = 5; 19.2%), vocal behavior of schoolchildren (n = 4; 15.4%), vocal complaints, vocal signs and symptoms (n = 3; 11.5%), vocal habits (n = 3; 11.5%), environmental characteristics (n = 2; 7.7%), interviews (n = 1; 3.8%), anthropometric data, health history and development of schoolchildren (n = 1; 3.8%), diseases and communication disorders (n = 1; 3.8%), and application of the MTA-SNAP-IV (n = 1) and the Brazilian National Economic Indicator (n = 1; 3.8%).

**Chart 2:** Actions developed according to the components of the School Health Program

Authors and year	Characteristics of actions according to SHP components
Cielo and Cappellari, 2008^(22)^	Component I – Health Assessment
	Parent Questionnaire on Child's General Health
	Auditory-Perceptual Evaluation (RASAT)
	Aerodynamic Assessment (Maximum Phonation Time /a/, /s/, /z/)
	Hearing Screening
Cappellari and Cielo, 2008^(23)^	Component I – Health Assessment
	Parent Questionnaire on Child's General Health
	Auditory-Perceptual Evaluation (RASAT)
	Acoustic Analysis (Multi-Dimensional Voice Program Advanced)
	Hearing Screening
Sales et al., 2010^(24)^	Component I – Assessment of health conditions
	Interview
	Vocal screening
	Auditory-Perceptual Evaluation (GRBAS)
	Assessment of other communication disorders during spontaneous conversation
	**At another institution**
	Otolaryngological evaluation (physical examination and video nasolaryngoscopy).
Silva et al., 2012^(25)^	Component I – Health Assessment
	Parent questionnaire on the child's general health and assessment of inappropriate vocal habits
	Vocal Screening (APE)
	Social Skills Assessment (Multimedia Inventory of Social Skills for Children - IMHSC)
Sales et al., 2013^(26)^	Component I – Assessment of Health Conditions
	Questionnaire for children about negative feelings when using their voice and vocal complaints
	Vocal screening with assessment of the following parameters: pitch (predominantly low or high), intensity (adequate, reduced, or increased), resonance (oral, nasal, or laryngeal), and speech rate (predominantly normal, slow, or fast).
	Aerodynamic assessment (maximum phonation time /a/, /i/, /u/, /s/, /z/)
	Acoustic analysis (Multi-Speech Program)
Sales et al., 2013^(27)^	Component I – Health Assessment
	Questionnaire for children about negative feelings when using their voice and vocal complaints
	Auditory-perceptual evaluation (GRBAS)
	Acoustic analysis (Multi-Speech program)
	Aerodynamic assessment (maximum phonation time /a/, /i/, /u/, /s/, /z/)
	Other aspects assessed during spontaneous conversation: word articulation, vocal attack, resonance, pitch, loudness, and breathing/speech coordination
	Conducting a game-based workshop for socialization
	**At another institution:**
	Otolaryngological evaluation (laryngeal video stroboscopy)
Pascotini et al., 2015^(28)^	Component I – Health Assessment
	Questionnaire for parents about the child's vocal behavior (vocal identity, favorite games, vocal habits and family environment, pathological factors, and parental behavior regarding the vocal change)
Pascotini et al., 2016^(29)^	Component I – Health Assessment
	Acoustic analysis (Multi-Dimensional Voice Program Advanced)
	Aerodynamic assessment (maximum inspiratory pressure and maximum expiratory pressure measured with a pressure gauge)
	Anthropometric measurements (weight and height) and calculation of Body Mass Index (BMI)
	Hearing screening
Marangon et al., 2018^(10)^	Component I – Health Assessment
	Auditory-Perceptual evaluation (GRBASI)
	Aerodynamic Assessment (breathing pattern, lip position during breathing, and nasal airflow measured using a Glatzel mirror)
Aires et al., 2019^(30)^	Component I – Health Assessment
	Parent Interview about the Child's General Health
	Auditory-Perceptual evaluation (CAPE-V)
	Aerodynamic Assessment (maximum phonation time /a/, /i/)
	Hearing Screening
Hoffmann and Cielo, 2021^(31)^	Component I – Health Assessment
	Parent Interview about anthropometric data, health history, and the child's development
	Auditory-Perceptual evaluation (CAPE-V);
	Aerodynamic Assessment (maximum phonation time /a/, /i/, /u/)
	Acoustic Analysis (Multi-Dimensional Voice Program Advanced)
	Hearing Screening
Schott et al., 2009^(32)^	Component I – Assessment of Health Conditions
	Screening (Adapted Form from the ENT and Head and Neck Surgery Clinic Assessment)
	Auditory-Perceptual evaluation (RASAT)
	Acoustic Analysis (VoxMetria)
Braga et al., 2009^(33)^	Component I – Health Assessment
	Child assessment form sent to parents
	Auditory-perceptual evaluation (RASAT)
	Acoustic analysis (VoxMetria)
Oliveira et al., 2011^(12)^	Component I – Health Assessment
	Auditory-perceptual evaluation (GRBASI)
	Acoustic analysis (Multi-Dimensional Voice Program Advanced)
	Feedback for the school and parents and/or guardians
	Guidance on vocal health
	Referral for care at the Basic Health Unit for children with dysphonia
Paixão et al., 2012^(34)^	Component I – Health Assessment
	Questionnaire with parents about habits that are harmful to the voice and characteristics of the environment
	Questionnaires with students to identify habits that are harmful to the voice and characteristics of the environment
	Auditory-perceptual evaluation (classification of altered/unaltered voice)
	Feedback for parents
Souza et al., 2017^(35)^	Component I – Health Assessment
	Parental vocal self-assessment (PVRQOL)
	Auditory-perceptual evaluation (overall degree of dysphonia graded on a 4-point Likert scale)
Silva and Souza, 2020^(36)^	Component I – Health Assessment
	Acoustic analysis (PRAAT program)
	Aerodynamic assessment (maximum phonation time /a/, /i/, /u/)
Reis et al., 2021^(37)^	Component I – Health Assessment
	Vocal self-assessment (PVRQOL)
	Acoustic analysis (VoxMetria program)
Tavares et al., 2011^(38)^	Component I – Health Assessment
	Questionnaires for parents about the child's general health, vocal complaints, and associated factors.
	Auditory-perceptual evaluation (GRBASI)
	Acoustic analysis (Multi-Dimensional Voice Program Advanced)
	**At another institution**
	Video laryngoscopy
Guerra et al., 2014^(39)^	Component I – Assessment of Health Conditions
	Questionnaire for parents on vocal behavior, vocal signs/symptoms, diseases, and communication disorders
	Questionnaire for the teacher on vocal behavior, vocal signs/symptoms, diseases, and communication disorders
	Guidance for families on vocal behavior changes and where children could be referred for treatment, when necessary
Paixão et al., 2015^(40)^	Component I – Assessment of Health Conditions
	Questionnaire for parents on vocal behavior, vocal/laryngeal signs and symptoms, and environmental characteristics
	Questionnaire for children on vocal behavior, vocal/laryngeal signs and symptoms, and environmental characteristics
	Auditory-Perceptual evaluation (GRBASI)
	Feedback for parents
	Guidance for families
	Referral of children with vocal disorders for otorhinolaryngological and speech-language pathology evaluation, as well as treatment, when necessary
Pascotini et al., 2014^(41)^	Component I – Assessment of Health Conditions
	Parental vocal self-assessment (P-QVV)
	Anthropometric assessment (weight and height) and calculation Body Mass Index (BMI)
Pascotini et al., 2016^(42)^	Component I – Health Assessment
	Aerodynamic assessment (Forced Vital Capacity by spirometry and Maximum Phonation Time of the vowels /a/, /e/, /ė/)
	Hearing screening
	Anthropometric assessment (weight and height) and calculation of Body Mass Index (BMI)
	Waist circumference measurement
Cielo et al., 2016^(43)^	Component I – Health Assessment
	Aerodynamic assessment (Maximum Phonation Time of the vowels /e/, /é/)
	Hearing screening
	Anthropometric assessment (weight and height) and calculation of BMI
	Pubertal development assessment through pediatric consultation
Maia, 2012^(44)^	Component I – Health Assessment
	Interview with parents about vocal behavior, speech characteristics, and vocal habits, and administration of the MTA-SNAP-IV questionnaire
	Teachers indicate children without complaints of academic or social impairment and analyze their academic performance
	Auditory-perceptual evaluation (Scale) GRBAS and Visual Analog Scale)
	Acoustic analysis (VoxMetria program)
	Global behavioral and speech-language-hearing assessment
	**At another institution**
	Otolaryngological evaluation (laryngeal evaluation by fiberoptic nasoscopy)
Nunes, 2017^(45)^	Component I – Health Assessment
	Questionnaire for parents on the National Economic Indicator (IEN)
	Parental vocal self-assessment (PVRQOL)
	Auditory-perceptual evaluation (GRBASI)
	Acoustic analysis (Multi-Dimensional Voice Program Advanced)
	Analysis of vocal behavior (vocal attack)
	Parental behavioral assessment (Child Behavior Checklist - CBCL)
Oliveira, 2023^(46)^	Component II – Health Promotion and Disease Prevention
	Stage 1
	Development and evaluation of the course on children's vocal health by experts and laypeople
	Stage 2 – Pilot Study
	Questionnaire for parents on Participant Profile Characterization – before and after
	Tests – before and after
	Online course for parents and students
	Stage 3 – Evaluation of the Online Course by more participants
	Questionnaire for parents on Participant Profile Characterization – before and after
	Tests – before and after
	Online course for parents and students

Some studies (n = 4; 15.4%) conducted questionnaires or interviews with the students to identify vocal complaints, signs, and symptoms (n = 3; 11.5%), environmental characteristics (n = 2; 7.7%), negative feelings when using the voice (n = 2; 7.7%), vocal behavior (n = 1; 3.8%), and vocal habits (n = 1; 3.8%).

Of the 26 studies that performed multidimensional assessment of the students' voices, 65.4% (n = 17) performed auditory-perceptual evaluation of the voice; 50% (n = 13) performed acoustic analysis; 38.5% (n = 10) performed aerodynamic assessment; 15.4% (n = 4) performed self-assessment; and 15.4% (n = 4) performed laryngological assessment – the latter at health services outside the school.

Other SLH aspects were evaluated (n = 10; 38.5%), including hearing screening (n = 7; 27%) and evaluation of other aspects of communication (n = 3; 11.5%). Complementary evaluations included anthropometric assessment with calculation of body mass index (BMI) (n = 4; 15.4%), evaluation of pubertal development (n = 1; 3.8%), measurement of abdominal circumference (n = 1; 3.8%), evaluation of social skills (n = 1; 3.8%), global behavior and SLH assessment (n = 1; 3.8%), and behavioral evaluation (n = 1; 3.8%).

Feedback on data obtained in assessments with students was reported in 11.5% (n = 3) of the studies; guidance to families, in 11.5% (n = 3); and referrals for assessment or treatment of the observed changes were mentioned in 11.5% (n = 3) of the studies that carried out assessments.

Of the 27 studies analyzed, 7.4% (n = 2) included procedures with teachers. One identified children with good academic performance, and the other administered a questionnaire on vocal behavior, vocal signs and symptoms, diseases, and communication disorders observed in schoolchildren.

A single study among the 27 analyzed involved health promotion and disease prevention initiatives and was developed in three stages^(46)^. The first stage consisted of the development and evaluation of a virtual course by experts and laypeople; the second stage was a pilot study with five parents and seven schoolchildren without vocal complaints; and the third stage involved implementing the program with 10 additional parents and 12 schoolchildren, also without vocal complaints. Stages 2 and 3 included, in addition to the online course for parents and schoolchildren, questionnaires to characterize the participants' profiles and pre- and post-course tests.

## DISCUSSION

Despite the existence of public policies that encourage an emphasis on preventive SLH measures, the small number of articles selected for this scoping review reflects the scarcity of contributions on ​​childhood voice conducted in public schools, even though the latter are an important setting for actions that address the three SHP components. Furthermore, the fact that 96.3% of the studies selected for this review involved only procedures for assessing students' health status corroborates this statement and raises another aspect to be discussed: the scarcity of scientific publications linked to public policies.

The results highlight the scarcity of studies involving schoolchildren. Only 14.8% of the articles were published in the last 5 years. The years with the highest publication frequency were 2012 and 2016, with three articles each. It is difficult to identify the reasons for the higher number of publications during this period, as we did not identify any public policy that might have encouraged publications or any specific line of research initiated during this period.

Some articles included hearing assessment^(22,23,29-31,42,43)^ of students and the evaluation of aspects related to nutrition, such as anthropometric assessment with BMI calculation and measurement of abdominal circumference^(29,41-43)^; these two actions are also covered in Article 4 of Decree No. 6,286^(7)^.

The analysis of the articles selected for this study showed a predominance of actions related to Component I of the SHP (n = 26; 96%), and only one article (4%) included actions provided for in Component II. We did not find any articles involving training actions related to Component III, which aims to train SHP young protagonists and education and health managers or professionals linked to the Program^(8,9)^.

One possible interpretation for this result could be that the researchers responsible for these articles were not officially affiliated with the SHP in the municipalities where they were conducted, or even that the municipality had not joined the Program.

Regarding the origin of the articles, there was a lack of studies conducted in Northern Brazil. The state with the highest frequency of articles was Rio Grande do Sul. Research on the profile of undergraduate SLH programs showed that the regions offering the fewest programs were the North (9.6%) and the Central-West (6%), and that some states (Tocantins, Amapá, and Roraima) did not offer undergraduate SLH programs^(47)^. The studies from the Southern Region were carried out by researchers from the Federal University of Santa Maria^(22,23,28-31,41-43)^, which has one of the oldest SLH programs in Brazil, making a significant contribution to the development of knowledge in the field.

There was also a higher frequency of articles published in national journals^(10,12,22,23,25,28,32-37,39-43)^ than in international ones^(24,26,27,29-31,38)^. This finding may have occurred due to the lesser interest of international journal editors in publishing the topic studied in this review – voice-based interventions with schoolchildren in the context of a Brazilian public policy.

Among the national journals, Revista CEFAC had the largest number of publications. Revista CEFAC^(48)^, published since 1999, appears to be the preferred choice for publications focusing on the interface between SLH Pathology and Education, likely reflecting the CEFAC institution, which identifies itself as an organization focused on Health and Education teaching and research, aiming to deepen professional knowledge and develop new opportunities for action.

The sample sizes of the studies varied across the articles. Because they did not present sample size calculations, it is impossible to infer whether they were sufficient to generalize the findings to the general population, and the studies may lack external validity. Furthermore, it is important to mention the likelihood of Type II error, as it may not be possible to identify statistical differences in outcomes where they exist due to insufficient sample size. Therefore, it is necessary to interpret the results of studies with small sample sizes cautiously.

Among all articles analyzed, we found more actions (92.6%) directly involving students in the research procedures^(10,12,22-27,29-38,40-46)^. Among the 27 studies, 63% involved parents in interviews or questionnaires completed at home to gather information about their students^(22,23,25,28,30-34,38-40,44-46)^ or participation in parental vocal assessment using the Pediatric Voice-Related Quality-of-Life Survey (PVRQOL)^(35,41)^. Only two studies (7.4%) included schoolteachers in the research procedures^(39,44)^. The predominance of children in the studies may be due not only to an interest in understanding childhood voice disorders but also to the ease of accessing them during school activities, without compromising academic routines. Furthermore, the presence of parents or guardians at school is often difficult due to work.

A study conducted to analyze SHP initiatives through individual semi-structured interviews with program coordinators in Porto Alegre, Rio Grande do Sul, showed that participants recognized the great potential of the prevention and health promotion work carried out within the context of this public policy. However, they highlighted some challenges, including the involvement of parents/families of schoolchildren. Among the 13 health professionals and coordinators interviewed, only one reported good family participation in the kindergarten meetings and activities proposed by the SHP. In the remaining interviews, the family-school partnership proved fragile; the coordinators reported attempts by the SHP to engage with the parents/families of schoolchildren but observed little adherence^(49)^.

The articles analyzed in this scoping review did not provide much information about parental compliance with researchers' invitations to participate in research activities or to provide feedback on the results of assessments conducted with students. However, in two studies, not all parents or guardians returned the questionnaires and authorizations sent – only 53% and 47% were returned^(28,34)^.

The studies focused on schoolchildren aged 4 to 12, with a predominance of research involving elementary school students^(10,12,24-38,40-46)^. Only five studies^(22,23,38,39,46)^ approached students from preschool and kindergarten, with children up to 5 years old.

The younger the child, the more important it is for the studies to include their parents and teachers, so that they can be raised and educated on health promotion and disease prevention by identifying habits, behaviors, and characteristics of the home or school environment that may contribute to the development of voice disorder^(10)^. Furthermore, the sooner a child is referred for evaluation and treatment, the less likely the consequences of the voice disorder will be for the child.

The literature highlights habits such as excessive talking, singing, and loud speaking as common in schoolchildren^(28,34)^. These inappropriate vocal habits, when practiced frequently and for a long time, can trigger voice disorders^(10,44)^. Inappropriate vocal behaviors, with voice abuse and misuse, are frequently observed in recreational activities at the school and home, in addition to the use of inappropriate vocal models, which contribute to the risk of voice disorders^(10,12,28,38-40,46)^. Due to the expected characteristics of a child's voice, parents may not notice signs suggestive of vocal changes in their children, which delays the search for specialized care^(28,31,34,35,38,40,45,46)^. Therefore, professionals working with schoolchildren must be aware of vocal changes, especially to guide parents and students on vocal habits and behaviors, with a view to maintaining vocal health.

A study conducted in two municipal preschools in Marília, São Paulo, which used a questionnaire with parents and assessed the vocal behavior of schoolchildren through observation of teaching activities, identified complaints suggestive of vocal changes in 11.84% of the students and frequent harmful voice habits adopted by 16.44%. Interviews with the schoolchildren in this study revealed limited knowledge about voice, highlighting the need for actions aimed at preventing dysphonia and promoting vocal health in the schools involved. Its authors proposed educational work on voice with preschoolers^(50)^.

Regarding the multidimensional assessment procedures of students' voices, the auditory-perceptual evaluation was most frequently performed^(10,12,22-24,27,30-35,38,40,44,45)^. It is the primary voice assessment tool in SLH clinics and is considered the gold standard for identifying voice disorders^(51)^. Because it is a quick, inexpensive, and easy-to-perform procedure, involving only on-site recording, it can be easily performed in schools. The only precaution required is that the students' voices be recorded in a quiet location to facilitate auditory-perceptual analysis by SLH pathologists experienced in voice.

It is noteworthy that, of the 26 studies that assessed the health status of schoolchildren, 61% involved questionnaires, interviews, or surveys with their parents or guardians. However, only 15.4% of the studies provided feedback or guidance to parents about voice or the results of the assessment procedures performed with the schoolchildren^(12,34,39,40)^; 7.7% referred for further assessments and/or treatment when necessary^(12,40)^; and 3.8%, although they did not make the referral, informed about places to which students could be referred for treatment when necessary^(39)^.

Contacting parents or guardians to provide information about research procedures or feedback on the results is an important opportunity to disseminate information about students' voices, potentially contributing to the promotion of vocal health and the prevention of voice disorders^(12,34,39,40)^. These actions are essential, since vocal changes often go unnoticed by parents, despite their identifying abusive vocal behaviors. When they do, they are frequently attributed to symptoms of upper respiratory tract infections (35). However, feedback or referrals do not constitute health education actions organized and carried out with the school community to promote health and prevent injuries, as proposed in Article 3 of Decree No. 6,286, which states that "the SHP is a strategy for the integration and permanent coordination between education and health policies and actions, with the participation of the school community, involving the family health and basic education teams"^(7)^.

Of the 18 studies that sent questionnaires to parents or guardians of schoolchildren^(22,23,25,28,30-35,37-41,44-46)^, only 11.5%^(35,37,41)^ used a parental vocal self-assessment protocol, the PVRQOL. The remaining questionnaires aimed to obtain data on vocal complaints, signs, symptoms, habits, behaviors, and characteristics of the schoolchildren's environment. Also, 23.1% of the studies^(24,26,27,34,40)^ used questionnaires with schoolchildren, and only one of them used the PVRQOL. According to the literature, children from 6 years old are already able to reflect on their vocal problems and present complaints about the symptoms they experience^(14)^.

Furthermore, it is known that children have greater awareness of their vocal symptoms and their influence on their daily lives than their parents. However, it is worth noting that PVRQOL is not recommended for children, as it was validated for use exclusively with parents, and there is no evidence of its effectiveness with children. Other validated protocols in Brazilian Portuguese have versions for self-perception and parental perception^(52,53)^.

This finding highlights the low use of vocal self-assessment questionnaires in the studies analyzed – multidimensional voice assessment by SLH pathologists was the least common. Due to the existence of validated instruments in Brazilian Portuguese that can be administered to both parents and children, and the fact that the literature indicates a score almost three times higher in children's self-perception compared to parental perception, their application is recommended for both populations in vocal screening and in SLH diagnosis and monitoring^(52,53)^.

Only 7.4% of the articles formally included the students’ teachers in the research procedures^(39,44)^. One included them to identify children with good academic performance, and the other to obtain information about the students' health. Disseminating knowledge about vocal health to teachers and other school staff members is of great importance, as they are in contact with students in their daily work and can contribute not only to the identification of voice disorders but also to the prevention of inappropriate vocal behaviors and the management of environmental issues that may contribute to the occurrence of these disorders. Furthermore, by helping them adopt healthy vocal habits and reduce noise in the school environment, these professionals will also be taking care of their own voices. Moreover, disseminating information about vocal health to teachers meets the proposals of SHP Component III.

Voice disorders may be more common in occupational voice users, such as teachers. The organization of the work process, the work environment, and individual characteristics are considered the three risk factors for occupational voice disorders (OVD). They help trigger and/or maintain vocal problems and symptoms, and, when related to the person's work activity, constitute OVD^(54)^, a highly prevalent health problem among teachers^(55)^. Therefore, investing in actions within Component III of the SHP may be one of the best vocal health strategies for teachers.

When carrying out actions on children’s voices in schools, it is necessary to adopt a broader and multifactorial view, investigating school routine procedures, environmental conditions, and their relationship with the schoolchildren’s vocal production, as well as actions aimed at raising awareness and providing clarification to parents, educators, and schoolchildren about the importance of voice in children's communication, learning, and socialization^(12)^.

Collective health promotion actions in schools, involving students, teachers, and staff, should contribute to developing each person's ability to interpret daily life and act to incorporate appropriate attitudes and behaviors to improve quality of life. Thus, health and education professionals must recurrently empower students, teachers, and school staff to embrace the basic principles of health promotion^(10)^.

Although this scoping review included studies conducted during the SHP's validity period, none of them reported being included in or even mentioned the publication of this interministerial public policy. Knowledge of public health and education policies is essential for SLH pathologists working in educational institutions to develop actions consistent with these guidelines.

It is also important to emphasize that the topic of this article (actions on voice in childhood) is not mentioned among the health actions provided for under the SHP and indicated in Article 4 of Decree No. 6,286, which established the Program^(7)^. However, more recent government publications related to this program include the "identification of schoolchildren with possible signs of oral language impairment" as a priority action from an epidemiological point of view, which can be developed with students in daycare, preschool, elementary school, and high school^(9)^. Although these documents do not mention voice, it is understood that vocal changes can compromise oral communication and child development.

The limitation of this review is the lack of consultation with an end user to review the data extraction protocol.

## CONCLUSION

In conclusion, studies on children’s vocal performance in Brazilian public schools are scarce. Existing ones prioritized the development of procedures related to component I of the SHP and aimed at assessing the vocal health of schoolchildren.

## Appendix A Search strategies for databases and gray literature

**Table:**

Databases	Strategy	Number of studies mapped
Medline/ PUBMED	(("Preschool children"[Title/Abstract] OR "Young children"[Title/Abstract] OR "Early childhood"[Title/Abstract] OR "Preschoolers"[Title/Abstract] OR "Pre-kindergarten children"[Title/Abstract] OR "Nursery school children"[Title/Abstract] OR "Pre-K children"[Title/Abstract] OR "Kindergarten children"[Title/Abstract] OR "Early learners"[Title/Abstract] OR "Toddlers"[Title/Abstract] OR "Children aged 3-5"[Title/Abstract] OR "Children aged 4-6"[Title/Abstract] OR "Early years children"[Title/Abstract] OR "Preprimary children"[Title/Abstract] OR "Infant school children"[Title/Abstract] OR "Children"[Title/Abstract] OR "Child"[Title/Abstract]) AND ("Voice"[Title/Abstract] OR "Phonation"[Title/Abstract] OR "Vocal function"[Title/Abstract] OR "Vocal quality"[Title/Abstract] OR "Voice production"[Title/Abstract] OR "Vocal health"[Title/Abstract] OR "Laryngeal function"[Title/Abstract] OR "Vocal fold function"[Title/Abstract] OR "Vocal cord function"[Title/Abstract] OR "Vocal performance"[Title/Abstract] OR "Vocal expression"[Title/Abstract]) AND ("Primary education"[Title/Abstract] OR "Elementary education"[Title/Abstract] OR "Secondary education"[Title/Abstract] OR "High school education"[Title/Abstract] OR "K-12 education"[Title/Abstract] OR "School education"[Title/Abstract] OR "Grade school education"[Title/Abstract] OR "Middle school education"[Title/Abstract] OR "Junior high school education"[Title/Abstract] OR "Primary schooling"[Title/Abstract] OR "Secondary schooling"[Title/Abstract] OR "Basic education"[Title/Abstract] OR "Compulsory education"[Title/Abstract] OR "Early education"[Title/Abstract] OR "School"[Title/Abstract]	354
LILACS/VHL	(TW:("crianças em idade pré-escolar" OR "crianças pré-escolares" OR "crianças pequenas" OR "primeira infância" OR "pré-escolares" OR "crianças em pré-jardim de infância" OR "crianças de escola infantil" OR "crianças de pré-K" OR "crianças de jardim de infância" OR "primeiros aprendizes" OR "crianças de 3-5 anos" OR "crianças de 4-6 anos" OR "crianças de primeiros anos" OR "crianças pré-primárias" OR "crianças de escola infantil" OR "crianças" OR "criança") AND TW:("voz" OR "fonação" OR "função vocal" OR "qualidade vocal" OR "produção vocal" OR "saúde vocal" OR "função laríngea" OR "função das pregas vocais" OR "função das cordas vocais" OR "performance vocal" OR "expressão vocal") AND TW:("educação primária e secundária" OR "educação primária" OR "educação fundamental" OR "educação secundária" OR "educação de ensino médio" OR "educação K-12" OR "educação escolar" OR "educação de ensino básico" OR "educação obrigatória" OR "educação inicial" OR "escola"))	86
EMBASE	('preschool child'/exp OR 'preschool child' OR 'young child':ab,ti OR 'early childhood':ab,ti OR 'preschooler':ab,ti OR 'pre-kindergarten child':ab,ti OR 'nursery school child':ab,ti OR 'pre-k child':ab,ti OR 'kindergarten child':ab,ti OR 'early learner':ab,ti OR 'toddler':ab,ti OR 'child aged 3-5':ab,ti OR 'child aged 4-6':ab,ti OR 'early years child':ab,ti OR 'preprimary child':ab,ti OR 'infant school child':ab,ti OR 'child':ab,ti) AND ('voice'/exp OR 'voice' OR 'phonation':ab,ti OR 'vocal function':ab,ti OR 'vocal quality':ab,ti OR 'voice production':ab,ti OR 'vocal health':ab,ti OR 'laryngeal function':ab,ti OR 'vocal fold function':ab,ti OR 'vocal cord function':ab,ti OR 'vocal performance':ab,ti OR 'vocal expression':ab,ti) AND ('primary education'/exp OR 'primary education' OR 'elementary education':ab,ti OR 'secondary education':ab,ti OR 'high school education':ab,ti OR 'k-12 education':ab,ti OR 'school education':ab,ti OR 'grade school education':ab,ti OR 'middle school education':ab,ti OR 'junior high school education':ab,ti OR 'primary schooling':ab,ti OR 'secondary schooling':ab,ti OR 'basic education':ab,ti OR 'compulsory education':ab,ti OR 'early education':ab,ti OR 'school':ab,ti)	337
SCOPUS	( ( TITLE-ABS-KEY ( "Preschool children" OR "Young children" OR "Early childhood" OR "Preschoolers" OR "Pre-kindergarten children" OR "Nursery school children" OR "Pre-K children" OR "Kindergarten children" OR "Early learners" OR "Toddlers" OR "Children aged 3-5" OR "Children aged 4-6" OR "Early years children" OR "Preprimary children" OR "Infant school children" ) ) AND ( TITLE-ABS-KEY ( "Voice" OR "Phonation" OR "Vocal function" OR "Vocal quality" OR "Voice production" OR "Vocal health" OR "Laryngeal function" OR "Vocal fold function" OR "Vocal cord function" OR "Vocal performance" OR "Vocal expression" ) ) AND ( TITLE-ABS-KEY ( "Education, Primary and Secondary" OR "Primary education" OR "Elementary education" OR "Secondary education" OR "High school education" OR "K-12 education" OR "School education" OR "Grade school education" OR "Middle school education" OR "Junior high school education" OR "Primary schooling" OR "Secondary schooling" OR "Basic education" OR "Compulsory education" OR "Early education" ) ) )	64
WEB OF SCIENCE	((TS=("Preschool children" OR "Young children" OR "Early childhood" OR "Preschoolers" OR "Pre-kindergarten children" OR "Nursery school children" OR "Pre-K children" OR "Kindergarten children" OR "Early learners" OR "Toddlers" OR "Children aged 3-5" OR "Children aged 4-6" OR "Early years children" OR "Preprimary children" OR "Infant school children")) AND TS=("Voice" OR "Phonation" OR "Vocal function" OR "Vocal quality" OR "Voice production" OR "Vocal health" OR "Laryngeal function" OR "Vocal fold function" OR "Vocal cord function" OR "Vocal performance" OR "Vocal expression")) AND TS=("Education, Primary and Secondary" OR "Primary education" OR "Elementary education" OR "Secondary education" OR "High school education" OR "K-12 education" OR "School education" OR "Grade school education" OR "Middle school education" OR "Junior high school education" OR "Primary schooling" OR "Secondary schooling" OR "Basic education" OR "Compulsory education" OR "Early education" OR "School")	165
Cochrane Central Register of Controlled Trials	(("Preschool children" OR "Young children" OR "Early childhood" OR "Preschoolers" OR "Pre-kindergarten children" OR "Nursery school children" OR "Pre-K children" OR "Kindergarten children" OR "Early learners" OR "Toddlers" OR "Children aged 3-5" OR "Children aged 4-6" OR "Early years children" OR "Preprimary children" OR "Infant school children" OR "Children" OR "Child")):ti,ab,kw AND (("Voice" OR "Phonation" OR "Vocal function" OR "Vocal quality" OR "Voice production" OR "Vocal health" OR "Laryngeal function" OR "Vocal fold function" OR "Vocal cord function" OR "Vocal performance" OR "Vocal expression")):ti,ab,kw AND (("Education, Primary and Secondary" OR "Primary education" OR "Elementary education" OR "Secondary education" OR "High school education" OR "K-12 education" OR "School education" OR "Grade school education" OR "Middle school education" OR "Junior high school education" OR "Primary schooling" OR "Secondary schooling" OR "Basic education" OR "Compulsory education" OR "Early education" OR "School")):ti,ab,kw	129
ProQuest	noft(("Preschool children" OR "Young children" OR "Early childhood" OR "Preschoolers" OR "Pre-kindergarten children" OR "Nursery school children" OR "Pre-K children" OR "Kindergarten children" OR "Early learners" OR "Toddlers" OR "Children aged 3-5" OR "Children aged 4-6" OR "Early years children" OR "Preprimary children" OR "Infant school children" OR "Children" OR "Child")) AND noft(("Voice" OR "Phonation" OR "Vocal function" OR "Vocal quality" OR "Voice production" OR "Vocal health" OR "Laryngeal function" OR "Vocal fold function" OR "Vocal cord function" OR "Vocal performance" OR "Vocal expression")) AND noft(("Education, Primary and Secondary" OR "Primary education" OR "Elementary education" OR "Secondary education" OR "High school education" OR "K-12 education" OR "School education" OR "Grade school education" OR "Middle school education" OR "Junior high school education" OR "Primary schooling" OR "Secondary schooling" OR "Basic education" OR "Compulsory education" OR "Early education" OR "School")) AND stype.exact("Dissertations & Theses")	86
MedRxiv	"Preschool children" AND "Voice" AND "Education, Primary and Secondary"	0
Google Scholar	“Voice” AND “Dysphonia” AND “School”	2,856
