# Supplementary material for: Actions on childhood voice in public schools: a scoping review
Source: Codas. 2026 Jan 26;38(1):e20240367. doi: 10.1590/2317-1782/e20240367en (PMC12978321; doi:10.1590/2317-1782/e20240367en)
Supplement: Translation

# Translation: Ações sobre voz na infância em escolas públicas: revisão de escopo

**DOI:** 10.1590/2317-1782/e20240367pt

**Authors:** Luciana Tavares Sebastião, Nelma Ellen Zamberlan-Amorim, Fabiana Zambon, Vanessa Veis Ribeiro, Mara Behlau

**Affiliations:** 1 Centro de Estudos da Voz – CEV - São Paulo (SP), Brasil.; 2 Hospital das Clínicas, Faculdade de Medicina de Ribeirão Preto - Ribeirão Preto (SP), Brasil.; 3 Sindicato dos Professores de São Paulo – SINPRO-SP - São Paulo (SP), Brasil.; 4 Faculdade de Ciências e Tecnologias em Saúde, Universidade de Brasília – UnB - Brasília (DF), Brasil.

**Keywords:** Voz, Distúrbio da Voz, Pré-escolar, Ensino Fundamental e Médio, Política Pública, Fonoaudiologia

## Abstract

**Objetivo:**

mapear ações sobre voz na infância desenvolvidas em escolas públicas brasileiras e analisá-las segundo componentes do Programa Saúde na Escola (PSE).

**Estratégia de pesquisa:**

trata-se de uma revisão de escopo, estruturada segundo as diretrizes metodológicas do Instituto Joanna Briggs (JBI) e que teve como pergunta norteadora: Quais ações sobre voz na infância são desenvolvidas em escolas públicas do Brasil e quais componentes do PSE estão contemplados nessas ações? Foi realizada busca eletrônica nas bases de dados MEDLINE, LILACS, Scopus, Web of Science, Embase e Cochrane, busca manual na literatura cinzenta, lista de citações e consulta aos especialistas.

As estratégias de busca foram compostas por termos livres e indexados relacionados ao tema e adaptados para cada fonte de evidências.

**Critérios de seleção:**

foram selecionados estudos envolvendo escolares até 12 anos de idade, ações sobre voz desenvolvidas por fonoaudiólogos, em escolas da rede pública de ensino e publicados após 2007. Foram extraídos dados da publicação, da amostra e ações sobre voz infantil segundo os componentes do PSE.

**Análise dos dados:**

a análise de dados foi descritiva.

**Resultados:**

foram encontrados 4.087 estudos e 27 foram selecionados. As ações envolveram os escolares, com maior frequência, seguidos pelos pais e professores; 26 estudos incluíram ações de avaliação das condições de saúde dos escolares e um, ações de promoção da saúde e prevenção de agravos em um curso online para pais e escolares. O procedimento de avaliação mais frequente foi o julgamento perceptivo-auditivo.

**Conclusão:**

estudos na área de voz na infância em escolas são escassos e priorizam avaliação das condições de saúde vocal.

## INTRODUÇÃO

Política pública é um conjunto de ações do governo com objetivos específicos, cujas atividades influenciam a vida dos cidadãos^(1)^. O direito à saúde alcança o cidadão por meio das políticas públicas que visam ao atendimento integral, com ênfase nas atividades preventivas e no controle social, bem como melhores indicadores de saúde^(2-4)^. Há um incentivo mundial para que maiores investimentos sejam destinados à promoção da saúde e ao aumento da qualidade de vida da população^(5)^.

A Fonoaudiologia corrobora com essa perspectiva, como profissão da saúde, por meio do planejamento recente da *American Speech-Language-Hearing Association* (ASHA). Esse planejamento soma esforços para que, até 2030, a profissão amplie sua ênfase em ações que visem a melhorar a qualidade de vida em populações saudáveis, atuando sobre os determinantes da saúde da comunicação e sobre o estilo de vida saudável relacionado à comunicação humana^(6)^.

O Programa Saúde na Escola (PSE) é uma política interministerial brasileira, instituída pelo Decreto Presidencial nº 6.286, de 5 de dezembro de 2007^(7)^. O PSE está atualmente estruturado em três componentes: Avaliação das condições de saúde dos escolares (Componente I); Promoção da saúde e prevenção de agravos (Componente II); e Formação (Componente III). Este último componente envolve a formação dos gestores do programa e das equipes de Educação e de Saúde e de Jovens Protagonistas para o PSE^(8,9)^.

No Brasil, estima-se que cerca de 12,8% das crianças matriculadas em escolas públicas do ensino básico apresentem distúrbio vocal^(10)^. Distúrbios vocais podem trazer impactos negativos para a qualidade de vida dos escolares por dificultarem a comunicação eficiente e a inserção nas atividades sociais e educacionais^(11,12)^.

A voz infantil possui peculiaridades decorrentes da imaturidade neuromuscular do aparelho fonador e do formato das cartilagens laríngeas. Características vocais como rouquidão, soprosidade e instabilidade leves são consideradas esperadas em crianças, em decorrência do ligamento vocal imaturo, da indiferenciação das camadas da prega vocal, da grande vascularização das pregas vocais com tendência a edema, do tamanho e posição das pregas vocais decorrentes do formato das cartilagens laríngeas na infância e da imaturidade neuromuscular. Na infância, a voz não se diferencia em função do gênero, podendo haver pequenas variações em função da ressonância no trato vocal^(13)^.

Escolares com distúrbios vocais podem apresentar queixas como rouquidão, dor, pigarro e tosse ao falar e cantar, dificuldades para ler em voz alta ou cantar em jogos e brincadeiras. Podem, ainda, apresentar frustração, raiva, constrangimento e insatisfação com a voz^(14)^. Apesar disso, apenas cerca de 23% das crianças com distúrbio vocal buscam atendimento fonoaudiológico^(15)^.

Esses dados confirmam a necessidade de ações do PSE voltadas à promoção da saúde vocal, bem como à prevenção, identificação, tratamento e monitoramento de distúrbios vocais na escola.

Nesse sentido, considera-se importante mapear as ações de voz infantil que vêm sendo desenvolvidas em escolas públicas do Brasil, além de classificá-las a partir dos componentes do PSE. Acredita-se que esses dados proporcionarão uma maior compreensão sobre o estado da arte da implementação do PSE junto aos escolares brasileiros e trarão evidências que poderão embasar discussões acerca dos limites e das necessidades de investimentos nessa área, visando a diminuir os riscos para o desenvolvimento e a melhorar a qualidade de vida desses escolares.

Estudos de revisão sobre ações em voz desenvolvidas com escolares de escolas públicas são bastante escassos e, em geral, buscam analisar aspectos vocais e comportamentais, não visando, como neste artigo, caracterizar as ações em voz desenvolvidas nas escolas considerando políticas públicas de saúde^(16,17)^.

O objetivo desta revisão de escopo foi mapear ações sobre voz na infância desenvolvidas em escolas públicas do Brasil e classificar os aspectos abordados de acordo com os componentes do PSE.

## MÉTODO

Trata-se de uma revisão de escopo que seguiu a metodologia do *Joanna Briggs Institute* ^(18)^ e foi escrita conforme as recomendações do PRISMA-ScR. O protocolo da presente revisão de escopo foi registrado na *Open Science Framework* (doi: 10.17605/OSF.IO/UE94W).

A pergunta norteadora da pesquisa seguiu o acrônimo P - Participante; C - Conceito; e C - Contexto (PCC), sendo: P - crianças; C - componentes do PSE presentes em ações sobre voz; e C - escolas públicas do Brasil. As perguntas de pesquisa que nortearam a revisão foram: Quais ações sobre voz na infância são desenvolvidas em escolas públicas do Brasil? Quais componentes do PSE estão contemplados nas ações?

A busca foi realizada de forma eletrônica e manual. A busca eletrônica foi conduzida nas bases de dados LILACS (BVS), MEDLINE (PubMed), EMBASE, Web of Science, SCOPUS e Cochrane Library. A busca manual foi realizada por meio do mapeamento de citações, da literatura cinzenta (Google Scholar, medRxiv e ProQuest) e da consulta a experts (mapeamento de publicações de autores correspondentes com mais de três artigos selecionados na busca eletrônica ou manual).

A estratégia de busca foi desenvolvida com base nos unitermos indexados (*Medical Subject Headings* – MeSH e *Emtree terms*) e termos livres relacionados ao PCC. Inicialmente, foi desenvolvida uma estratégia de busca para a MEDLINE, posteriormente adaptada para as demais fontes de evidência (Apêndice A).

Foram selecionados estudos envolvendo escolares de até 12 anos de idade, com ações sobre voz desenvolvidas por fonoaudiólogos em escolas da rede pública de ensino brasileira, e publicados após 2007.

Para a seleção dos estudos, foram estabelecidos critérios de inclusão e exclusão. Foram incluídos artigos que envolveram escolares de zero a 12 anos de idade, ações sobre voz infantil realizadas por fonoaudiólogos e desenvolvidas em escolas da rede pública de ensino do Brasil. Foram excluídos estudos com delineamento de revisão de literatura, anais de eventos e Trabalhos de Conclusão de Curso de Graduação; artigos sobre ações desenvolvidas com voz de adolescentes, adultos ou idosos (idade > 12 anos); estudos com amostras compostas apenas por escolares da rede particular de ensino; estudos realizados em outros países que não o Brasil; estudos duplicados; estudos com amostras duplicadas; e estudos aceitos para publicação antes de 2007. O filtro do ano de publicação foi utilizado porque a data de instituição do Programa Saúde na Escola foi dezembro de 2007. Não foi utilizado filtro de idioma. O corte de idade em 12 anos teve como base a Lei nº 8.069, de 13 de julho de 1990, que dispõe sobre o Estatuto da Criança e do Adolescente, e considera como criança a pessoa até doze anos de idade incompletos^(19)^.

A seleção dos estudos seguiu três etapas: remoção dos duplicados; leitura de título e resumo, com aplicação dos critérios de inclusão; e leitura do texto completo, com aplicação dos critérios de exclusão. A seleção na busca eletrônica foi realizada no site Rayyan. Esse processo será descrito nos resultados, utilizando-se o fluxograma de seleção do PRISMA-ScR. Dois autores independentes realizaram a seleção dos estudos, e as discordâncias foram resolvidas por consenso.

Uma calibração prévia foi realizada entre os revisores antes do início da seleção, utilizando-se 25 artigos. Para serem aprovados na calibração, os revisores precisaram obter um coeficiente de concordância no Kappa de Cohen de, no mínimo, 0,7. A calibração foi realizada, e foi obtido um coeficiente de concordância de 1,0. Valores de Kappa de Cohen entre 0,81 e 1,0 indicam concordância quase perfeita^(20)^. A busca foi realizada em junho de 2024, e a seleção foi conduzida entre junho e julho de 2024.

Para a extração de dados, os autores elaboraram um instrumento específico para este estudo. Foram extraídos dados sobre: publicação (autores, ano de publicação, estado em que o estudo foi realizado, periódico); características da amostra (tamanho da amostra, nível e tipo de ensino, idade e gênero dos participantes); e características das ações desenvolvidas nas escolas, segundo os componentes do PSE (avaliação das condições de saúde; promoção da saúde e prevenção de agravos; e formação). As ações presentes nos estudos selecionados foram classificadas pelos autores da presente revisão de escopo, de acordo com a descrição dos três componentes atuais do PSE^(8,9)^.

A extração de dados foi realizada por dois revisores independentes. As discordâncias foram resolvidas por consenso. Caso fosse necessária a obtenção de dados adicionais ou a confirmação de informações, estavam previstas até três tentativas de contato com o autor correspondente, via e-mail.

Os dados foram analisados de forma descritiva, por frequência simples e relativa. Os resultados serão apresentados no formato de quadros, gráficos, tabelas e figuras.

## RESULTADOS

A Figura 1 apresenta os estudos identificados nas buscas eletrônica e manual. Na busca eletrônica, foram inicialmente identificados 1.135 estudos. Desse total, 222 estudos foram removidos por duplicação, e 897 não atenderam aos critérios de inclusão. Durante a primeira fase de seleção, 16 artigos foram incluídos com base na leitura de título e resumo. Na segunda fase, cinco artigos foram excluídos: três porque os procedimentos não foram realizados em escolas da rede pública; um por apresentar apenas o diagnóstico situacional da escola; e um por envolver banco de dados de vozes de professores. Dessa forma, foram selecionados 11 estudos na busca eletrônica.

**Figura 1:** Fluxograma de seleção dos estudos

Fonte: Page MJ, et al.^(21)^. Traduzido pelos autores

**Legenda:** n=frequência absoluta; TCC=Trabalho de Conclusão de Curso

Na busca manual, foram identificados 2.856 estudos no Google Scholar e 86 no ProQuest. Desses, 30 foram incluídos com base na leitura de título e resumo. Na leitura completa, 20 estudos foram excluídos: seis por se tratar de Trabalhos de Conclusão de Curso; quatro por incluírem escolares com idades superiores a 12 anos; cinco por não terem sido realizados com escolares de escolas públicas; dois por serem publicados em anais de eventos; dois por apresentarem apenas o desenvolvimento de material educativo; e um por não ter sido realizado por fonoaudiólogo. Assim, foram selecionados dez estudos nesta fase.

Ainda na busca manual, foram incluídos três estudos por meio do mapeamento de citações e três por meio da consulta a experts.

Dessa forma, foram selecionados para esta revisão de escopo 27 estudos, sendo 24 artigos e três teses de doutorado.

Com relação às características de publicação (Tabela 1), 14,8% (n = 4) dos estudos foram publicados nos últimos cinco anos (2020 a 2024). Os anos com maior frequência de publicações foram 2012 e 2016 (n = 3; 11,1% cada). Os estudos são oriundos de grupos de pesquisa de quatro regiões e oito estados brasileiros, principalmente da região Sul do Brasil (n = 12; 44,4%) e do estado do Rio Grande do Sul (n = 9; 33,3%). Dentre as 24 publicações em formato de artigo, 75% (n = 18) foram publicadas em periódicos nacionais, com maior frequência na *Revista CEFAC* (n = 7; 38,9%).

**Tabela 1:** Características de publicação

Autores	Estado	Ano	Periódico
Cielo e Cappellari^(22)^	RS	2008	Revista Brasileira de Otorrinolaringologia
Cappellari e Cielo^(23)^	RS	2008	Revista Brasileira de Otorrinolaringologia
Sales et al.^(24)^	SE	2010	*Journal of Voice*
Silva et al.^(25)^	PR	2012	Jornal da Sociedade Brasileira de Fonoaudiologia
Sales et al.^(26)^	SE	2013	*Journal of Voice*
Sales et al.^(27)^	SE	2013	*Journal of Voice*
Pascotini et al.^(28)^	RS	2015	Distúrbios da Comunicação
Pascotini et al.^(29)^	RS	2016	*Journal of Voice*
Marangon et al.^(10)^	SP	2018	Revista CEFAC
Aires et al.^(30)^	RS	2019	*Journal of Voice*
Hoffmann e Cielo^(31)^.	RS	2021	*Journal of Voice*
Schott et al.^(32)^	RJ	2009	Revista CEFAC
Braga et al.^(33)^	MG	2009	Revista CEFAC
Oliveira et al.^(12)^	MG	2011	Jornal da Sociedade Brasileira de Fonoaudiologia
Paixão et al.^(34)^	PR	2012	Revista CEFAC
Souza et al.^(35)^	MG	2017	CoDAS
Silva and Souza^(36)^	RN	2020	Revista Brasileira de Ciências da Saúde
Reis et al.^(37)^	PE	2021	Revista CEFAC
Tavares et al.^(38)^	SP	2011	*Brazilian Journal of Otorhinolaryngology*
Guerra et al.^(39)^	PE	2014	Distúrbios da Comunicação
Paixão et al.^(40)^	PR	2015	Distúrbios da Comunicação
Pascotini et al.^(41)^	RS	2014	Revista Brasileira de Qualidade de Vida
Pascotini et al.^(42)^	RS	2016	Revista CEFAC
Cielo et al.^(43)^	RS	2016	Revista CEFAC
Maia^(44)^	MG	2012	Tese de doutorado (UFMG)
Nunes^(45)^	MG	2017	Tese de doutorado (UFMG)
Oliveira^(46)^	SP	2023	Tese de doutorado (Unesp Marília)

Com relação às características da amostra (Quadro 1), houve a presença de escolares em 92,6% (n = 25) dos estudos, de pais em 33,3% (n = 9) e de professores em 7,4% (n = 2). O tamanho da amostra variou entre 15 e 2.000 escolares, e entre 15 e 198 pais. Com relação ao número de professores, um dos estudos incluiu um professor e o outro não informou o número de professores participantes. A faixa etária dos alunos variou de quatro a 12 anos, sendo que 85,2% (n = 23) dos estudos incluíram escolares de ambos os sexos. Vale destacar que 14,8% (n = 4) dos estudos não informaram o sexo dos participantes. Foram mais frequentes os estudos com escolares do nível de ensino Fundamental (n = 22; 81,5%). No tocante ao tipo de instituição educacional, predominaram os estudos com escolares exclusivamente da rede pública de ensino (n = 20; 74%). No entanto, alguns estudos compuseram suas amostras com escolares do ensino público e privado (n = 7; 26%).

**Quadro 1:** Características da amostra

Autores e ano	Participantes			Tamanho da Amostra	Idade (anos)	Sexo	Nível de ensino	Rede de Ensino
	Crianças	Pais	Professores					
Cielo e Cappellari, 2008^(22)^	**º**			23 crianças	4:0 a 6:8	Ambos os sexos	Educação Infantil	Pública Privada
Cappellari e Cielo, 2008^(23)^	**º**			23 crianças	4:0 a 6:8	Ambos os sexos	Educação Infantil	Pública Privada
Sales et al., 2010^(24)^	**º**			600 crianças	7 a 10	Ambos os sexos	Ensino Fundamental	Pública
Silva et al., 2012^(25)^	**º**			38 crianças	7 a 11	Ambos os sexos	Ensino Fundamental	Pública
Sales et al., 2013^(26)^	**º**			196 crianças	7 a 10	Ambos os sexos	Ensino Fundamental	Pública
Sales et al., 2013^(27)^	**º**			60 crianças	7 a 10	Ambos os sexos	Ensino Fundamental	Pública
Pascotini et al., 2015^(28)^		**º**		104 pais	8 a 12	Ambos os sexos	Ensino Fundamental	Pública
Pascotini et al., 2016^(29)^	**º**			82 crianças	8 a 10	Ambos os sexos	Ensino Fundamental	Pública
Marangon et al., 2018^(10)^	**º**			250 crianças	6 a 9	Ambos os sexos	Ensino Fundamental	Pública
Aires et al., 2019^(30)^	**º**			154 crianças	8 a 10	Ambos os sexos	Ensino Fundamental	Pública Privada
Hoffmann e Cielo, 2021^(31)^	**º**			115 crianças	4:7 a 7:11	Não informado	Ensino Fundamental	Pública Privada
Schott et al., 2009^(32)^	**º**			122 crianças	6:0 a 8:11	Ambos os sexos	Ensino Fundamental	Pública
Braga et al., 2009^(33)^	**º**			100 crianças	6 a 8	Ambos os sexos	Ensino Fundamental	Pública
Oliveira et al., 2011^(12)^	**º**			70 crianças	6 a 10	Ambos os sexos	Ensino Fundamental	Pública
Paixão et al., 2012^(34)^	**º**	**º**		50 crianças 100 pais	6 a 12	Ambos os sexos	Ensino Fundamental	Pública
Souza et al., 2017^(35)^	**º**			420 crianças	6 a 10	Não informado	Ensino Fundamental	Pública Privada
Silva; Souza, 2020^(36)^	**º**			63 crianças	8 a 12	Ambos os sexos	Ensino Fundamental	Pública
Reis et al., 2021^(37)^	**º**			31 crianças	6 a 7	Ambos os sexos	Ensino Fundamental	Pública
Tavares et al., 2011^(38)^	**º**			2.000 crianças	4 a 12	Ambos os sexos	Educação Infantil Ensino Fundamental	Pública Privada
Guerra et al., 2014^(39)^		**º**	**º**	20 pais 1 professora	4 a 6	Ambos os sexos	Educação Infantil	Pública
Paixão et al., 2015^(40)^	**º**	**º**		50 crianças 100 pais	6 a 12	Não informado	Ensino Fundamental	Pública
Pascotini et al., 2014^(41)^	**º**	**º**		83 crianças	8 a 12	Ambos os sexos	Ensino Fundamental	Pública
Pascotini et al., 2016^(42)^	**º**			82 crianças	8 a 10	Ambos os sexos	Ensino Fundamental	Pública
Cielo et al., 2016^(43)^	**º**	**º**		102 crianças	8 a 12	Ambos os sexos	Ensino Fundamental	Pública
Maia, 2012^(44)^	**º**	**º**	**º**	198 crianças 198 pais professores NI	7 a 10	Ambos os sexos	Ensino Fundamental	Pública
Nunes, 2017^(45)^	**º**	**º**		420 crianças	6 a 10	Ambos os sexos	Ensino Fundamental	Pública Privada
Oliveira, 2023^(46)^	**º**	**º**		19 crianças 15 pais	4 a 11	Não informado	Educação Infantil Ensino Fundamental	Pública

Apenas dois dos três componentes do PSE estiveram presentes nos estudos, com destaque para o Componente I — avaliação das condições de saúde dos educandos (Figura 2).

**Figura 2:** Caracterização dos componentes do PSE abordados nos estudos

Observa-se no Quadro 2 que, dentre os 26 estudos que realizaram avaliação das condições de saúde dos escolares, 46,2% (n = 12) envolveram questionários ou entrevistas com pais ou responsáveis pelos alunos para levantamento de informações sobre a saúde geral dos escolares (n = 5; 19,2%), comportamento vocal dos escolares (n = 4; 15,4%), queixas vocais, sinais e sintomas vocais (n = 3; 11,5%), hábitos vocais (n = 3; 11,5%), características do ambiente (n = 2; 7,7%), entrevistas (n = 1; 3,8%), dados antropométricos, histórico de saúde e desenvolvimento do escolar (n = 1; 3,8%), doenças e alterações de comunicação (n = 1; 3,8%), aplicação do questionário MTA-SNAP-IV (n = 1) e do Indicador Econômico Nacional (n = 1; 3,8%).

**Quadro 2:** Ações desenvolvidas segundo os componentes do PSE

Autores e ano	Características das ações segundo os componentes do PSE
Cielo e Cappellari, 2008^(22)^	Componente I – Avaliação das condições de saúde
	Questionário para pais sobre saúde geral da criança
	Julgamento perceptivo-auditivo (RASAT)
	Avaliação aerodinâmica (tempo máximo de fonação /a/, /s/, /z/)
	Triagem auditiva
Cappellari e Cielo, 2008^(23)^	Componente I – Avaliação das condições de saúde
	Questionário para pais sobre saúde geral da criança
	Julgamento perceptivo-auditivo (RASAT)
	Análise acústica (programa*Multi-Dimensional Voice Program Advanced)*
	Triagem auditiva
Sales et al., 2010^(24)^	Componente I – Avaliação das condições de saúde
	Entrevista
	Triagem vocal
	Julgamento perceptivo-auditivo (GRBAS)
	Avaliação de outros distúrbios da comunicação durante conversa espontânea
	**Em outra instituição**
	Avaliação otorrinolaringológica (exame físico e videonasolaringoscopia).
Silva et al., 2012^(25)^	Componente I – Avaliação das condições de saúde
	Questionário para pais sobre saúde geral da criança e levantamento de hábitos vocais inadequados
	Triagem vocal (JPA)
	Avaliação das habilidades sociais (Inventário Multimídia de Habilidades Sociais para Crianças - IMHSC)
Sales et al., 2013^(26)^	Componente I – Avaliação das condições de saúde
	Questionário para crianças sobre sentimentos negativos ao usar a voz e queixas vocais
	Triagem vocal com avaliação dos seguintes parâmetros: altura (predominantemente baixa ou alta), intensidade (adequada, reduzida ou aumentada), ressonância (oral, nasal ou laríngea) e taxa de fala (predominantemente normal, lenta ou rápida).
	Avaliação aerodinâmica (tempo máximo de fonação /a/, /i/, /u/, /s/, /z/
	Análise acústica (Programa Multi-Speech)
Sales et al., 2013^(27)^	Componente I – Avaliação das condições de saúde
	Questionário para crianças sobre sentimentos negativos ao usar a voz e queixas vocais
	Julgamento perceptivo-auditivo (GRBAS)
	Análise acústica (programa Multi-Speech)
	Avaliação aerodinâmica (tempo máximo de fonação /a/, /i/, /u/, /s/, /z/)
	Outros aspectos avaliados durante conversa espontânea: articulação das palavras, ataque vocal, ressonância, *pitch, loudness*, coordenação pneumofônica
	Realização de um workshop baseado em jogos para socialização
	**Em outra instituição:**
	Avaliação otorrinolaringológica (videolaringoestroboscopia)
Pascotini et al., 2015^(28)^	Componente I – Avaliação das condições de saúde
	Questionário para pais sobre comportamento vocal da criança (identidade vocal, brincadeira predileta, hábitos vocais e ambiente familiar, fator patológico e comportamento dos pais com relação à alteração vocal)
Pascotini et al., 2016^(29)^	Componente I – Avaliação das condições de saúde
	Análise acústica (*Multi-Dimensional Voice Program Advanced)*
	Avaliação aerodinâmica (pressão inspiratória máxima e pressão expiratória máxima avaliadas com manômetro)
	Medidas antropométricas (peso e altura) e cálculo do Índice de Massa Corporal (IMC)
	Triagem auditiva
Marangon et al., 2018^(10)^	Componente I – Avaliação das condições de saúde
	Julgamento perceptivo-auditivo (GRBASI)
	Avaliação aerodinâmica (modo respiratório, posição dos lábios durante a respiração e fluxo nasal medido por meio do espelho de Glatzel)
Aires et al., 2019^(30)^	Componente I – Avaliação das condições de saúde
	Entrevista para pais sobre saúde geral da criança
	Julgamento perceptivo-auditivo (CAPE-V)
	Avaliação aerodinâmica (tempo máximo de fonação /a/, /i/)
	Triagem auditiva
Hoffmann e Cielo, 2021^(31)^	Componente I – Avaliação das condições de saúde
	Entrevista com pais sobre dados antropométricos, histórico de saúde e desenvolvimento da criança
	Julgamento perceptivo-auditivo (CAPE-V);
	Avaliação aerodinâmica (tempo máximo de fonação /a/, /i/, /u/)
	Análise acústica (programa*Multi-Dimensional Voice Program Advanced)*
	Triagem auditiva
Schott et al., 2009^(32)^	Componente I – Avaliação das condições de saúde
	Triagem (Formulário Adaptado da Avaliação da Clínica de ORL e Cirurgia de Cabeça e Pescoço)
	Julgamento perceptivo-auditivo (RASAT)
	Análise acústica (Voxmetria).
Braga et al., 2009^(33)^	Componente I – Avaliação das condições de saúde
	Formulário de avaliação da criança enviado para os pais
	Julgamento perceptivo-auditivo (RASAT)
	Análise acústica (Voxmetria)
Oliveira et al., 2011^(12)^	Componente I – Avaliação das condições de saúde
	Julgamento perceptivo-auditivo (GRBASI)
	Análise acústica (programa *Multi-Dimensional Voice Program Advanced*)
	Devolutiva para a escola e pais e/ou responsáveis
	Orientação sobre saúde vocal
	Encaminhamento para atendimento na Unidade Básica de Saúde das crianças disfônicas
Paixão et al., 2012^(34)^	Componente I – Avaliação das condições de saúde
	Questionário com os pais sobre hábitos prejudiciais à voz e características do ambiente
	Questionários com os alunos para identificar hábitos prejudiciais à voz e características do ambiente
	Julgamento perceptivo-auditivo (classificação em voz alterada/não alterada)
	Devolutiva para os pais
Souza et al., 2017^(35)^	Componente I – Avaliação das condições de saúde
	Autoavaliação vocal parental (QVV-P)
	Julgamento perceptivo-auditivo (grau geral de disfonia graduado em escala *Likert* de quatro pontos)
Silva e Souza, 2020^(36)^	Componente I – Avaliação das condições de saúde
	Análise acústica (programa PRAAT)
	Avaliação aerodinâmica (tempo máximo de fonação /a/, /i/, /u/)
Reis et al., 2021^(37)^	Componente I – Avaliação das condições de saúde
	Autoavaliação vocal (QVV-P)
	Análise acústica (programa VoxMetria)
Tavares et al., 2011^(38)^	Componente I – Avaliação das condições de saúde
	Questionários para os pais sobre saúde geral da criança, queixas vocais e fatores associados.
	Julgamento perceptivo-auditivo (GRBASI)
	Análise acústica (programa*Multi-Dimensional Voice Program Advanced*)
	**Em outra instituição**
	Videolaringoscopia
Guerra et al., 2014^(39)^	Componente I – Avaliação das condições de saúde
	Questionário para pais sobre comportamento vocal, sinais/sintomas vocais, doenças e alterações de comunicação
	Questionário para a professora sobre comportamento vocal, sinais/sintomas vocais, doenças e alterações de comunicação
	Orientações às famílias sobre alterações do comportamento vocal e local para o qual as crianças poderiam ser encaminhadas para tratamento, quando necessário
Paixão et al., 2015^(40)^	Componente I – Avaliação das condições de saúde
	Questionário para pais sobre comportamentos vocal, sinais e sintomas vocais/laríngeos e características do ambiente
	Questionário para crianças sobre comportamentos vocal, sinais e sintomas vocais/laríngeos e características do ambiente
	Julgamento perceptivo-auditivo (GRBASI)
	Devolutiva para pais
	Orientação às famílias
	Encaminhamento das crianças com alterações vocais para avaliação otorrinolaringológica e fonoaudiológica, bem como tratamento, quando necessário
Pascotini et al., 2014^(41)^	Componente I – Avaliação das condições de saúde
	Autoavaliação vocal parental (QVV-P)
	Avaliação antropométrica (peso e estatura) e cálculo do Índice de Massa Corporal (IMC)
Pascotini et al., 2016^(42)^	Componente I – Avaliação das condições de saúde
	Avaliação aerodinâmica (Capacidade Vital Forçada por espirometria e Tempo Máximo de Fonação das vogais /a/, /e/, /ė/)
	Triagem auditiva
	Avaliação antropométrica (peso e estatura) e cálculo do Índice de Massa Corporal (IMC)
	Medida da circunferência abdominal
Cielo et al., 2016^(43)^	Componente I – Avaliação das condições de saúde
	Avaliação aerodinâmica (Tempo Máximo de Fonação das vogais /e/, /é/)
	Triagem auditiva
	Avaliação antropométrica (peso e altura) e cálculo do IMC
	Avaliação desenvolvimento puberal por meio de consulta pediátrica
Maia, 2012^(44)^	Componente I – Avaliação das condições de saúde
	Entrevista com os pais sobre comportamento vocal, características da fala e hábitos vocais e aplicação do questionário MTA-SNAP-IV
	Indicação, pelas professoras, das crianças sem queixas de comprometimento acadêmico e social e análise do desempenho acadêmico
	Julgamento perceptivo-auditivo (Escala GRBAS e Escala Analógica Visual)
	Análise acústica (Programa VoxMetria)
	Avaliação comportamental global e fonoaudiológica
	**Em outra instituição**
	Avaliação otorrinolaringológica (avaliação laríngea por nasofibroscopia)
Nunes, 2017^(45)^	Componente I – Avaliação das condições de saúde
	Questionário para pais sobre Indicador Econômico Nacional (IEN)
	Autoavaliação vocal parental (QVV-P)
	Julgamento perceptivo-auditivo (GRBASI)
	Análise acústica (programa*Multi-Dimensional Voice Program Advanced)*
	Análise do comportamento vocal (ataque vocal)
	Avaliação comportamental parental *(Child Behavior Checklist* - CBCL)
Oliveira, 2023^(46)^	Componente II – Promoção da Saúde e Prevenção de Agravos
	Etapa 1
	Elaboração e avaliação do curso sobre saúde vocal infantil por especialistas e leigos
	Etapa 2 - Estudo Piloto
	Questionário para pais sobre Caracterização do Perfil do Participante – antes e após
	Provas – antes e após
	Curso *on-line* para pais e escolares
	Etapa 3 - Avaliação do Curso*On-line*por mais participantes
	Questionário para pais sobre Caracterização do Perfil do Participante – antes e após
	Provas – antes e após
	Curso *on-line* para pais e escolares

Alguns estudos (n = 4; 15,4%) realizaram questionários ou entrevistas com os próprios escolares, visando ao levantamento de queixas, sinais e sintomas vocais (n = 3; 11,5%), características do ambiente (n = 2; 7,7%), sentimentos negativos ao usar a voz (n = 2; 7,7%), comportamento vocal (n = 1; 3,8%) e hábitos vocais (n = 1; 3,8%).

Dos 26 estudos que realizaram avaliação multidimensional da voz dos escolares, 65,4% (n = 17) realizaram julgamento perceptivo-auditivo da voz; 50% (n = 13), análise acústica; 38,5% (n = 10), avaliação aerodinâmica; 15,4% (n = 4), autoavaliação; e 15,4% (n = 4), avaliação laringológica. A avaliação laringológica foi realizada em serviços de saúde externos à escola.

Outros aspectos fonoaudiológicos foram avaliados (n = 10; 38,5%), contemplando triagem auditiva (n = 7; 27%) e avaliação de outros aspectos da comunicação (n = 3; 11,5%). Avaliações complementares incluíram avaliação antropométrica com cálculo do Índice de Massa Corporal (IMC) (n = 4; 15,4%), avaliação do desenvolvimento puberal (n = 1; 3,8%), medida da circunferência abdominal (n = 1; 3,8%), avaliação das habilidades sociais (n = 1; 3,8%), avaliação do comportamento global e fonoaudiológico (n = 1; 3,8%) e avaliação comportamental (n = 1; 3,8%).

Devolutivas sobre os dados obtidos nas avaliações realizadas com os escolares foram relatadas em 11,5% (n = 3) dos estudos; orientação às famílias, em 11,5% (n = 3); e encaminhamentos para avaliação ou tratamento das alterações observadas foram mencionados em 11,5% (n = 3) dos estudos que realizaram avaliação.

Dentre os 27 estudos analisados, 7,4% (n = 2) incluíram procedimentos com professores, sendo um deles para a indicação de crianças com bom desempenho escolar e o outro para o preenchimento de um questionário sobre comportamento vocal, sinais/sintomas vocais, doenças e alterações de comunicação observadas nos escolares.

Um único estudo dentre os 27 analisados envolveu ações de promoção da saúde e prevenção de agravos e foi desenvolvido em três etapas^(46)^. A primeira etapa consistiu na elaboração e avaliação de um curso virtual por especialistas e leigos; a segunda etapa foi um estudo piloto com cinco pais e sete escolares sem queixas vocais; e a terceira etapa correspondeu à aplicação da ação com mais dez pais e 12 escolares também sem queixas vocais. As etapas 2 e 3 incluíram, além do curso online para pais e escolares, a aplicação de questionários para caracterização do perfil dos participantes e provas antes e após o curso virtual.

## DISCUSSÃO

Apesar da existência de políticas públicas que incentivam a ênfase em ações preventivas na Fonoaudiologia, o reduzido número de artigos selecionados para esta revisão de escopo reflete a escassez de contribuições na área de voz na infância realizadas em escolas públicas, mesmo com instituições da rede pública de ensino sendo um importante cenário para a realização de ações que contemplem os três componentes do PSE. Além disso, o fato de 96,3% dos estudos selecionados para esta revisão envolverem apenas procedimentos de avaliação das condições de saúde dos educandos corrobora essa afirmativa e leva a outro aspecto a ser discutido: a escassez de publicações científicas articuladas a políticas públicas.

Os resultados apontam a escassez de estudos com escolares. Apenas 14,8% dos artigos foram publicados nos últimos cinco anos. Os anos com maior frequência de publicações foram 2012 e 2016, com três artigos em cada ano. É difícil identificar as razões que expliquem um maior número de publicações nesse período, pois não identificamos nenhuma política pública que possa ter incentivado as publicações ou alguma linha de pesquisa específica iniciada nesse intervalo.

Alguns artigos incluíram a avaliação auditiva^(22,23,29-31,42,43)^ dos escolares e a avaliação de aspectos relacionados à nutrição, como avaliação antropométrica com cálculo do Índice de Massa Corporal e medida da circunferência abdominal^(29,41-43)^, sendo essas duas ações também contempladas no artigo 4º do Decreto nº 6.286^(7)^.

A análise dos artigos selecionados para este estudo mostrou predominância de ações relativas ao Componente I do PSE (n = 26; 96%) e apenas um artigo (4%) incluiu ações previstas no Componente II. Não encontramos nenhum artigo que envolvesse ações de formação relativas ao Componente III, que visa à formação de Jovens Protagonistas para o PSE e de Gestores ou Profissionais da Educação e da Saúde vinculados ao Programa^(8,9)^.

Uma das possíveis interpretações para esse resultado pode ser atribuída ao fato de os pesquisadores responsáveis pelos artigos analisados não estarem oficialmente vinculados ao PSE nos municípios em que foram realizados ou, até mesmo, ao fato de o município não ter feito a adesão ao Programa.

Com relação à procedência dos artigos, verificou-se a ausência de estudos realizados na região Norte do país. O estado com maior frequência de artigos foi o Rio Grande do Sul. Pesquisa sobre o perfil dos cursos de graduação em Fonoaudiologia mostrou que as regiões com menor oferta de cursos eram a Região Norte (9,6%) e a Centro-Oeste (6%), bem como que alguns estados (Tocantins, Amapá e Roraima) não possuíam curso de graduação em Fonoaudiologia^(47)^. Os estudos advindos da Região Sul foram realizados por pesquisadores da Universidade Federal de Santa Maria^(22,23,28-31,41-43)^, que conta com um dos cursos de Fonoaudiologia mais antigos do Brasil, com grande contribuição para a produção de conhecimento na área.

Verificou-se, ainda, maior frequência de artigos publicados em periódicos nacionais^(10,12,22,23,25,28,32-37,39-43)^, quando comparados aos periódicos internacionais^(24,26,27,29-31,38)^. Tal dado pode ter ocorrido devido ao menor interesse dos editores das revistas internacionais na publicação da temática estudada nesta revisão, a saber, ações em voz realizadas com escolares no contexto de uma política pública brasileira.

Dentre os periódicos nacionais, a *Revista CEFAC* foi a que teve o maior número de publicações. A *Revista CEFAC* ^(48)^, publicada desde 1999, parece ser a escolha para produções que focam na interface entre a Fonoaudiologia e a Educação, provável reflexo da instituição CEFAC, que se identifica como sendo uma organização voltada para o ensino e a pesquisa na área da Saúde e da Educação, tendo em vista o aprofundamento de conhecimentos profissionais e o desenvolvimento de novas possibilidades de atuação.

O tamanho amostral dos estudos foi variado nos diferentes artigos. Pelo fato de os estudos não apresentarem cálculo amostral, não há como inferir se são suficientes para a generalização dos achados para a população, podendo não haver validade externa nos estudos. Além disso, é importante citar a chance de erro tipo II, podendo não ser possível apontar diferenças estatísticas em desfechos nos quais elas existem, devido ao tamanho amostral insuficiente. Dessa forma, é necessário interpretar com cautela os resultados de estudos com baixo tamanho amostral encontrados.

Dentre o total de artigos analisados, verificamos maior número de ações (92,6%) envolvendo diretamente os escolares nos procedimentos de pesquisa^(10,12,22-27,29-38,40-46)^. Dentre os 27 estudos analisados, 63% envolveram os pais em entrevistas ou questionários respondidos em casa para o levantamento de informações sobre os escolares^(22,23,25,28,30-34,38-40,44-46)^ ou participação na avaliação vocal parental por meio do protocolo de Qualidade de Vida em Voz Pediátrico (QVV-P)^(35,41)^. Apenas dois estudos (7,4%) incluíram os professores dos escolares nos procedimentos de pesquisa realizados^(39,44)^. É possível que a predominância de crianças envolvidas nos estudos se deva não só ao interesse na compreensão de alterações vocais na infância, como também à facilidade de acesso a elas durante as atividades escolares, sem que a rotina acadêmica seja comprometida. Além disso, a presença de pais ou responsáveis na escola muitas vezes se torna difícil por motivos de trabalho.

Estudo realizado com o objetivo de analisar ações do PSE, por meio de entrevistas semiestruturadas individuais com coordenadores desse programa em Porto Alegre/RS, mostrou que os participantes reconheceram o grande potencial do trabalho de prevenção e promoção da saúde realizado no contexto dessa política pública. No entanto, apontaram alguns desafios, dentre eles o envolvimento dos pais/família dos escolares. Dentre os 13 profissionais de saúde e coordenadores entrevistados, apenas um relatou boa adesão dos familiares nas reuniões da escola infantil e nas atividades propostas pelo PSE. Nas demais entrevistas, a parceria família-escola se mostrou frágil; os coordenadores relataram tentativas de ações do PSE com os pais/famílias dos escolares, mas perceberam pouca adesão^(49)^.

Nos artigos analisados nesta revisão de escopo, não encontramos muitas informações sobre a adesão dos pais aos convites dos pesquisadores para as ações, visando fornecer informações sobre a pesquisa ou à realização das devolutivas sobre os resultados das avaliações feitas com os escolares. No entanto, em dois estudos foi possível perceber que nem todos os pais ou responsáveis devolveram os questionários e autorizações enviados; o retorno desses instrumentos foi de 53% e 47%^(28,34)^.

Os estudos se concentraram em escolares de quatro a 12 anos, com predominância de pesquisas com alunos do Ensino Fundamental^(10,12,24-38,40-46)^. Apenas cinco estudos^(22,23,38,39,46)^ envolveram escolares da Educação Infantil, que atende crianças com até cinco anos de idade.

Quanto menor a criança, maior a importância de que os estudos incluam seus pais e professores, para que eles sejam sensibilizados e orientados sobre promoção da saúde e prevenção de agravos, por meio da identificação de hábitos, comportamentos e características do ambiente residencial ou escolar que possam contribuir para o desenvolvimento de um distúrbio vocal^(10)^. Além disso, quanto antes ocorrer o encaminhamento para avaliação e tratamento, menores serão as consequências do distúrbio vocal para a criança.

Hábitos como falar muito, cantar e falar alto são apontados na literatura como frequentes em escolares^(28,34)^. Esses hábitos vocais inadequados, quando praticados com alta frequência e por longo prazo, podem desencadear o aparecimento de distúrbios vocais^(10,44)^. Comportamentos vocais inadequados, com abuso e mau uso da voz, são frequentemente observados em atividades recreativas no ambiente escolar e residencial, além do uso de modelos vocais inadequados, que contribuem para o risco de distúrbios vocais^(10,12,28,38-40,46)^. Devido às características esperadas na voz infantil, os pais podem não perceber os sinais sugestivos de alterações vocais em seus filhos, o que retarda a busca por atendimento especializado^(28,31,34,35,38,40,45,46)^. Assim, torna-se essencial que profissionais que trabalham com escolares estejam atentos às alterações vocais, principalmente no sentido de orientar os pais e os próprios escolares sobre hábitos e comportamentos vocais, com vistas à manutenção da saúde vocal.

Estudo realizado em duas escolas municipais de Educação Infantil de Marília/SP, que utilizou questionário com os pais e avaliou o comportamento vocal dos escolares por meio de observação em atividades didáticas, identificou queixas sugestivas da ocorrência de alterações vocais em 11,84% dos escolares e hábitos nocivos à voz adotados frequentemente por 16,44% deles. Neste estudo, entrevistas realizadas com os escolares revelaram escassos conhecimentos sobre voz, evidenciando a necessidade de se desenvolver ações voltadas à prevenção de disfonia e à promoção da saúde vocal nas escolas envolvidas. Nesta publicação, os autores apresentaram uma proposta de trabalho educativo sobre voz com escolares da Educação Infantil^(50)^.

Com relação aos procedimentos de avaliação multidimensional da voz dos alunos, foi mais frequente a realização do Julgamento Perceptivo-Auditivo (JPA)^(10,12,22-24,27,30-35,38,40,44,45)^. O JPA se configura como o principal instrumento da avaliação da voz na clínica fonoaudiológica, sendo considerado o padrão-ouro da avaliação vocal para a identificação de distúrbios vocais^(51)^. Por tratar-se de um procedimento rápido, barato e de fácil execução, que envolve apenas a etapa de gravação *in loco*, ele pode ser facilmente realizado em escolas. O único cuidado necessário é que a gravação das vozes dos escolares seja realizada em local silencioso, para viabilizar a análise perceptivo-auditiva por fonoaudiólogos com experiência na área de voz.

Chama atenção o fato de que, dentre os 26 estudos que realizaram avaliação das condições de saúde dos escolares, 61% envolveram questionários, entrevistas ou procedimentos de pesquisa com seus pais ou responsáveis. No entanto, apenas 15,4% dos estudos mencionaram a realização de devolutivas ou orientações aos pais sobre voz ou sobre os resultados dos procedimentos de avaliação realizados com os escolares^(12,34,39,40)^; 7,7% relataram a realização de encaminhamentos para avaliações complementares e/ou tratamento, quando necessário^(12,40)^; e 3,8%, embora não tenham realizado o encaminhamento, forneceram orientação sobre locais para os quais os escolares poderiam ser encaminhados para tratamento, quando necessário^(39)^.

O contato com os pais ou responsáveis, seja para fornecer informações sobre os procedimentos da pesquisa ou para a devolutiva sobre os resultados obtidos, é uma oportunidade importante para a disseminação de informações sobre a voz do escolar, com potencial para contribuir para a promoção da saúde vocal e para a prevenção de distúrbios da voz^(12,34,39,40)^. Essas ações são essenciais, visto que, muitas vezes, as alterações vocais não são percebidas pelos pais, apesar de eles identificarem comportamentos vocais abusivos, e, quando o são, frequentemente são atribuídas a sintomas de infecções das vias aéreas superiores^(35)^. No entanto, devolutivas ou encaminhamentos não se configuram como ações de educação em saúde organizadas e realizadas com a comunidade escolar com o objetivo de promoção da saúde e prevenção de agravos, conforme proposto no Artigo 3º do Decreto nº 6.286, ao indicar que “o PSE constitui estratégia para a integração e a articulação permanente entre as políticas e ações de educação e de saúde, com a participação da comunidade escolar, envolvendo as equipes de saúde da família e da educação básica”^(7)^.

Do total de 18 estudos que enviaram questionários para pais ou responsáveis pelos escolares^(22,23,25,28,30-35,37-41,44-46)^, apenas 11,5% deles^(35,37,41)^ utilizaram protocolos de autoavaliação vocal parental, sendo utilizado o QVV-P. Os demais questionários visaram à obtenção de dados sobre queixas vocais, sinais, sintomas, hábitos, comportamentos e características do ambiente dos escolares. Com relação a questionários com os escolares, 23,1% dos estudos^(24,26,27,34,40)^ utilizaram esse tipo de instrumento, sendo que apenas um deles utilizou o QVV-P. Segundo a literatura, crianças a partir dos seis anos de idade já são capazes de refletir sobre seus problemas vocais e apresentar queixas sobre os sintomas que vivenciam^(14)^.

Além disso, sabe-se que crianças têm maior percepção dos seus sintomas vocais e da influência deles em seu dia a dia do que seus pais. Porém, vale destacar que o QVV-P não é recomendado para ser respondido por crianças, pois foi validado para uso exclusivo com os pais e não há evidências de seu funcionamento com crianças. Na literatura, encontram-se outros protocolos validados em português brasileiro que têm versões de autopercepção e de percepção parental^(52,53)^.

Esse dado evidencia a baixa utilização de questionários de autoavaliação vocal nos estudos analisados, sendo o procedimento de avaliação multidimensional da voz realizado pelo fonoaudiólogo o menos frequente. Devido à existência de instrumentos validados em português brasileiro que possibilitam a aplicação tanto em pais quanto em crianças, bem como pelo fato de a literatura apontar um escore quase três vezes maior na autopercepção das crianças em relação à percepção parental, indica-se sua aplicação em ambas as populações, tanto em ações de triagem vocal quanto no diagnóstico e monitoramento do tratamento fonoaudiológico^(52,53)^.

Apenas 7,4% dos artigos incluíram formalmente os professores dos escolares estudados nos procedimentos de pesquisa^(39,44)^, sendo que um deles os incluiu para a indicação de crianças com bom desempenho escolar e o outro para obtenção de informações sobre a saúde dos escolares. Disseminar conhecimentos sobre saúde vocal para os professores e demais membros da equipe escolar tem grande importância, uma vez que esses profissionais estão em contato com os escolares no cotidiano do trabalho escolar e podem contribuir não só com a identificação de distúrbios vocais, mas também com a prevenção de comportamentos vocais inadequados e com o manejo de questões ambientais que possam favorecer a ocorrência desses distúrbios. Além disso, ao contribuir para a adoção de hábitos vocais saudáveis e para a redução do ruído no ambiente escolar, esses profissionais estarão também cuidando de sua própria voz. Ressalta-se, ainda, que a disseminação de informações sobre saúde vocal aos professores atende ao proposto no Componente III do PSE.

Distúrbios vocais podem ser mais frequentes na população que utiliza a voz profissionalmente, como os professores. Fatores relacionados à organização do processo de trabalho, ao ambiente de trabalho e às características individuais são considerados os três eixos de risco para distúrbios vocais relacionados ao trabalho. Esses eixos contribuem para o aparecimento e/ou manutenção de queixas e sintomas vocais e, estando relacionados à atividade laboral do indivíduo, configuram-se como Distúrbio de Voz Relacionado ao Trabalho (DVRT)^(54)^. O DVRT é um agravo à saúde de alta prevalência entre professores^(55)^. Dessa forma, entende-se que investir em ações do Componente 3 do PSE pode ser uma das melhores estratégias de saúde vocal para a população de professores.

Ao realizar ações sobre voz na infância em escolas, faz-se necessário adotar um olhar mais amplo e multifatorial, incluindo procedimentos de investigação das rotinas escolares, das condições ambientais e de sua relação com as condições de produção vocal dos escolares, assim como ações voltadas à conscientização e esclarecimentos aos pais, educadores e aos próprios escolares sobre a importância da voz na comunicação, no aprendizado e na socialização infantil^(12)^.

Ações coletivas de promoção da saúde em escolas, envolvendo escolares, professores e funcionários, devem contribuir para desenvolver, em cada um, a capacidade de interpretar o cotidiano e atuar de modo a incorporar atitudes e/ou comportamentos adequados para a melhoria da qualidade de vida. Nesse contexto, profissionais da Saúde e da Educação devem assumir uma atitude permanente de empoderamento dos princípios básicos de promoção da saúde por parte dos escolares, professores e funcionários das escolas^(10)^.

É importante salientar que, apesar de terem sido incluídos nesta revisão de escopo trabalhos realizados durante a vigência do PSE, nenhum dos artigos analisados descreveu estar inserido ou sequer mencionou a publicação dessa política pública interministerial. Ter conhecimento sobre as Políticas Públicas de Saúde e de Educação é fundamental para que, ao atuar em instituições educacionais, o fonoaudiólogo desenvolva ações coerentes com tais diretrizes.

É importante salientar, ainda, que o tema deste artigo, a saber, ações sobre voz na infância, não está citado entre as ações em saúde previstas no âmbito do PSE e indicadas no artigo 4º do Decreto nº 6.286, que instituiu o Programa^(7)^. No entanto, publicações governamentais mais recentes, relativas a este programa, incluem a “identificação de escolares com possíveis sinais de alteração da linguagem oral” como sendo ações prioritárias do ponto de vista epidemiológico, que podem ser desenvolvidas com escolares da Creche, Pré-Escola, Ensino Fundamental e Ensino Médio^(9)^. Embora a voz não seja citada em tais documentos, entende-se que alterações vocais podem comprometer a comunicação oral e o desenvolvimento infantil.

A presente revisão apresenta como limitação a ausência da consulta a um usuário final para a revisão do protocolo de extração de dados.

## CONCLUSÃO

Conclui-se que estudos sobre a atuação com voz na infância em escolas públicas brasileiras são escassos. Quando realizados, priorizam o desenvolvimento de procedimentos relacionados ao componente I do PSE e voltados à avaliação das condições de saúde vocal dos escolares.

## Apêndice A Estratégias de busca para as bases de dados e literatura cinzenta

**Table:**

Base de dados	Estratégia	Número de estudos mapeados
Medline/ PUBMED	(("Preschool children"[Title/Abstract] OR "Young children"[Title/Abstract] OR "Early childhood"[Title/Abstract] OR "Preschoolers"[Title/Abstract] OR "Pre-kindergarten children"[Title/Abstract] OR "Nursery school children"[Title/Abstract] OR "Pre-K children"[Title/Abstract] OR "Kindergarten children"[Title/Abstract] OR "Early learners"[Title/Abstract] OR "Toddlers"[Title/Abstract] OR "Children aged 3-5"[Title/Abstract] OR "Children aged 4-6"[Title/Abstract] OR "Early years children"[Title/Abstract] OR "Preprimary children"[Title/Abstract] OR "Infant school children"[Title/Abstract] OR "Children"[Title/Abstract] OR "Child"[Title/Abstract]) AND ("Voice"[Title/Abstract] OR "Phonation"[Title/Abstract] OR "Vocal function"[Title/Abstract] OR "Vocal quality"[Title/Abstract] OR "Voice production"[Title/Abstract] OR "Vocal health"[Title/Abstract] OR "Laryngeal function"[Title/Abstract] OR "Vocal fold function"[Title/Abstract] OR "Vocal cord function"[Title/Abstract] OR "Vocal performance"[Title/Abstract] OR "Vocal expression"[Title/Abstract]) AND ("Primary education"[Title/Abstract] OR "Elementary education"[Title/Abstract] OR "Secondary education"[Title/Abstract] OR "High school education"[Title/Abstract] OR "K-12 education"[Title/Abstract] OR "School education"[Title/Abstract] OR "Grade school education"[Title/Abstract] OR "Middle school education"[Title/Abstract] OR "Junior high school education"[Title/Abstract] OR "Primary schooling"[Title/Abstract] OR "Secondary schooling"[Title/Abstract] OR "Basic education"[Title/Abstract] OR "Compulsory education"[Title/Abstract] OR "Early education"[Title/Abstract] OR "School"[Title/Abstract]	354
LILACS/BV	(TW:("crianças em idade pré-escolar" OR "crianças pré-escolares" OR "crianças pequenas" OR "primeira infância" OR "pré-escolares" OR "crianças em pré-jardim de infância" OR "crianças de escola infantil" OR "crianças de pré-K" OR "crianças de jardim de infância" OR "primeiros aprendizes" OR "crianças de 3-5 anos" OR "crianças de 4-6 anos" OR "crianças de primeiros anos" OR "crianças pré-primárias" OR "crianças de escola infantil" OR "crianças" OR "criança") AND TW:("voz" OR "fonação" OR "função vocal" OR "qualidade vocal" OR "produção vocal" OR "saúde vocal" OR "função laríngea" OR "função das pregas vocais" OR "função das cordas vocais" OR "performance vocal" OR "expressão vocal") AND TW:("educação primária e secundária" OR "educação primária" OR "educação fundamental" OR "educação secundária" OR "educação de ensino médio" OR "educação K-12" OR "educação escolar" OR "educação de ensino básico" OR "educação obrigatória" OR "educação inicial" OR "escola"))	86
EMBASE	('preschool child'/exp OR 'preschool child' OR 'young child':ab,ti OR 'early childhood':ab,ti OR 'preschooler':ab,ti OR 'pre-kindergarten child':ab,ti OR 'nursery school child':ab,ti OR 'pre-k child':ab,ti OR 'kindergarten child':ab,ti OR 'early learner':ab,ti OR 'toddler':ab,ti OR 'child aged 3-5':ab,ti OR 'child aged 4-6':ab,ti OR 'early years child':ab,ti OR 'preprimary child':ab,ti OR 'infant school child':ab,ti OR 'child':ab,ti) AND ('voice'/exp OR 'voice' OR 'phonation':ab,ti OR 'vocal function':ab,ti OR 'vocal quality':ab,ti OR 'voice production':ab,ti OR 'vocal health':ab,ti OR 'laryngeal function':ab,ti OR 'vocal fold function':ab,ti OR 'vocal cord function':ab,ti OR 'vocal performance':ab,ti OR 'vocal expression':ab,ti) AND ('primary education'/exp OR 'primary education' OR 'elementary education':ab,ti OR 'secondary education':ab,ti OR 'high school education':ab,ti OR 'k-12 education':ab,ti OR 'school education':ab,ti OR 'grade school education':ab,ti OR 'middle school education':ab,ti OR 'junior high school education':ab,ti OR 'primary schooling':ab,ti OR 'secondary schooling':ab,ti OR 'basic education':ab,ti OR 'compulsory education':ab,ti OR 'early education':ab,ti OR 'school':ab,ti)	337
SCOPUS	( ( TITLE-ABS-KEY ( "Preschool children" OR "Young children" OR "Early childhood" OR "Preschoolers" OR "Pre-kindergarten children" OR "Nursery school children" OR "Pre-K children" OR "Kindergarten children" OR "Early learners" OR "Toddlers" OR "Children aged 3-5" OR "Children aged 4-6" OR "Early years children" OR "Preprimary children" OR "Infant school children" ) ) AND ( TITLE-ABS-KEY ( "Voice" OR "Phonation" OR "Vocal function" OR "Vocal quality" OR "Voice production" OR "Vocal health" OR "Laryngeal function" OR "Vocal fold function" OR "Vocal cord function" OR "Vocal performance" OR "Vocal expression" ) ) AND ( TITLE-ABS-KEY ( "Education, Primary and Secondary" OR "Primary education" OR "Elementary education" OR "Secondary education" OR "High school education" OR "K-12 education" OR "School education" OR "Grade school education" OR "Middle school education" OR "Junior high school education" OR "Primary schooling" OR "Secondary schooling" OR "Basic education" OR "Compulsory education" OR "Early education" ) ) )	64
WEB OF SCIENCE	((TS=("Preschool children" OR "Young children" OR "Early childhood" OR "Preschoolers" OR "Pre-kindergarten children" OR "Nursery school children" OR "Pre-K children" OR "Kindergarten children" OR "Early learners" OR "Toddlers" OR "Children aged 3-5" OR "Children aged 4-6" OR "Early years children" OR "Preprimary children" OR "Infant school children")) AND TS=("Voice" OR "Phonation" OR "Vocal function" OR "Vocal quality" OR "Voice production" OR "Vocal health" OR "Laryngeal function" OR "Vocal fold function" OR "Vocal cord function" OR "Vocal performance" OR "Vocal expression")) AND TS=("Education, Primary and Secondary" OR "Primary education" OR "Elementary education" OR "Secondary education" OR "High school education" OR "K-12 education" OR "School education" OR "Grade school education" OR "Middle school education" OR "Junior high school education" OR "Primary schooling" OR "Secondary schooling" OR "Basic education" OR "Compulsory education" OR "Early education" OR "School")	165
Cochrane Central Register of Controlled Trials	(("Preschool children" OR "Young children" OR "Early childhood" OR "Preschoolers" OR "Pre-kindergarten children" OR "Nursery school children" OR "Pre-K children" OR "Kindergarten children" OR "Early learners" OR "Toddlers" OR "Children aged 3-5" OR "Children aged 4-6" OR "Early years children" OR "Preprimary children" OR "Infant school children" OR "Children" OR "Child")):ti,ab,kw AND (("Voice" OR "Phonation" OR "Vocal function" OR "Vocal quality" OR "Voice production" OR "Vocal health" OR "Laryngeal function" OR "Vocal fold function" OR "Vocal cord function" OR "Vocal performance" OR "Vocal expression")):ti,ab,kw AND (("Education, Primary and Secondary" OR "Primary education" OR "Elementary education" OR "Secondary education" OR "High school education" OR "K-12 education" OR "School education" OR "Grade school education" OR "Middle school education" OR "Junior high school education" OR "Primary schooling" OR "Secondary schooling" OR "Basic education" OR "Compulsory education" OR "Early education" OR "School")):ti,ab,kw	129
ProQuest	noft(("Preschool children" OR "Young children" OR "Early childhood" OR "Preschoolers" OR "Pre-kindergarten children" OR "Nursery school children" OR "Pre-K children" OR "Kindergarten children" OR "Early learners" OR "Toddlers" OR "Children aged 3-5" OR "Children aged 4-6" OR "Early years children" OR "Preprimary children" OR "Infant school children" OR "Children" OR "Child")) AND noft(("Voice" OR "Phonation" OR "Vocal function" OR "Vocal quality" OR "Voice production" OR "Vocal health" OR "Laryngeal function" OR "Vocal fold function" OR "Vocal cord function" OR "Vocal performance" OR "Vocal expression")) AND noft(("Education, Primary and Secondary" OR "Primary education" OR "Elementary education" OR "Secondary education" OR "High school education" OR "K-12 education" OR "School education" OR "Grade school education" OR "Middle school education" OR "Junior high school education" OR "Primary schooling" OR "Secondary schooling" OR "Basic education" OR "Compulsory education" OR "Early education" OR "School")) AND stype.exact("Dissertations & Theses")	86
MedRxiv	"Preschool children" AND "Voice" AND "Education, Primary and Secondary"	0
Google Scholar	“Voice” AND “Dysphonia” AND “School”	2.856
